# Opisthobranchia (Mollusca, Gastropoda) – more than just slimy slugs. Shell reduction and its implications on defence and foraging

**DOI:** 10.1186/1742-9994-2-3

**Published:** 2005-02-16

**Authors:** Heike Wägele, Annette Klussmann-Kolb

**Affiliations:** 1 Museum König Bonn, Adenaueralle 160, 53113 Bonn, Germany; 2 Institut für Evolutionsbiologie, Rheinische Friedrich-Wilhelms-Universität, An der Immenburg 1, 53121 Bonn, Germany; 3 Zoologisches Institut, J.W. Goethe Universität, Siesmayerstrasse 70, 60054 Frankfurt, Germany

## Abstract

**Background:**

In general shell-less slugs are considered to be slimy animals with a rather dull appearance and a pest to garden plants. But marine slugs usually are beautifully coloured animals belonging to the less-known Opisthobranchia. They are characterized by a large array of interesting biological phenomena, usually related to foraging and/or defence. In this paper our knowledge of shell reduction, correlated with the evolution of different defensive and foraging strategies is reviewed, and new results on histology of different glandular systems are included.

**Results:**

Based on a phylogeny obtained by morphological and histological data, the parallel reduction of the shell within the different groups is outlined. Major food sources are given and glandular structures are described as possible defensive structures in the external epithelia, and as internal glands.

**Conclusion:**

According to phylogenetic analyses, the reduction of the shell correlates with the evolution of defensive strategies. Many different kinds of defence structures, like cleptocnides, mantle dermal formations (MDFs), and acid glands, are only present in shell-less slugs. In several cases, it is not clear whether the defensive devices were a prerequisite for the reduction of the shell, or reduction occurred before. Reduction of the shell and acquisition of different defensive structures had an implication on exploration of new food sources and therefore likely enhanced adaptive radiation of several groups.

## Background

**Figure 1:**
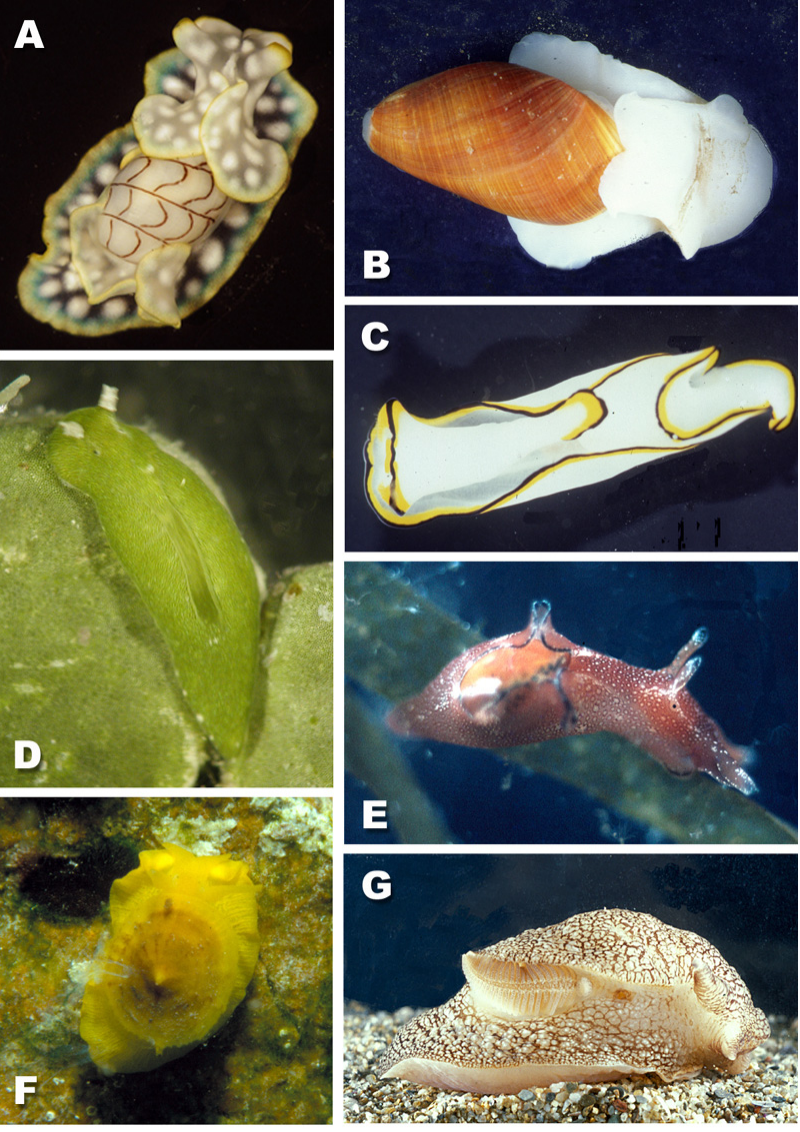
**Examples of opisthobranch species. **A *Micromelo undata *(Bruguière, 1792) (Acteonoidea) – Queensland, Australia, B *Scaphander lignarius *(Linné, 1758) (Cephalaspidea) – Northern Sea, C *Chelidonura pallida *Risbec, 1951 (Cephalaspidea) – Queensland, Australia, D *Elysiella pusilla *Bergh, 1872 (Sacoglossa) from the Indo Pacific, feeding on the green alga *Halimeda*; due to incorporation of chloroplasts, *Elysiella *has the same colour as the algae, E *Aplysia punctata *(Cuvier, 1803) (Anaspidea) – Mediterranean Sea, F *Tylodina perversa *(Gmelin, 1791) (Tylodinoidea) – Mediterranean Sea, G *Pleurobranchaea meckelii *Meckel in Leue, 1813 Mediterranean Sea.

Very often, non-shelled gastropods are considered to be slimy and non-attractive. This connotation usually refers to terrestrial species of the Stylommatophora belonging to the well-known Limacidae or Arionidae and in particular to garden snails, which do have negative effects on our horticulture. However, the Opisthobranchia are beautifully coloured "slimy" gastropods and exclusively occur in marine habitats. Non-scientists only meet these animals while diving for pleasure. One group of opisthobranchs, however, has become very famous throughout natural and even medical sciences: *Aplysia californica *Cooper, 1863, the sea hare, belonging to the subgroup Anaspidea (Fig. 1E). It is a classic example for neurobiological investigations, involving behaviour. It was E.R. Kandel, who performed many of his investigations on learning and memory on this animal [1]. He created the basic understanding of nerve functioning and learning in human beings and was awarded the noble prize of medicine in 2000 for his life time research on these animals.

**Figure 2:**
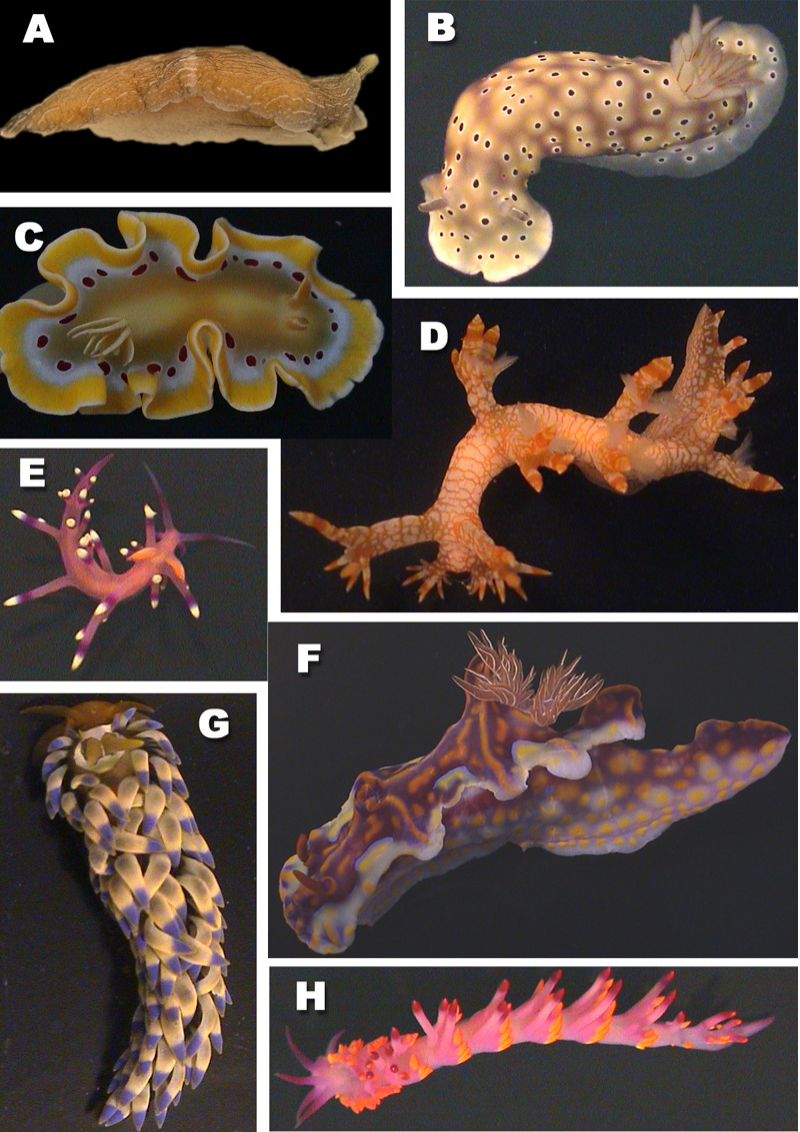
**Examples of nudibranch species. **A *Armina neapolitana *(Delle Chiaje, 1824) (Cladobranchia) – Mediterranean Sea, B *Risbecia tryoni *(Garrett, 1873) (Anthobranchia) – Queensland, Australia, C *Glossodoris cruenta *Rudman, 1986 (Anthobranchia) – Queensland, Australia, D *Bornella stellifer *(Adams & Reeve in Adams, 1848) (Cladobranchia) – Queensland, Australia, E *Flabellina exoptata *Gosliner & Willan, 1991 (Cladobranchia) – Queensland, Australia, F *Ceratosoma magnifica *(Van Hasselt, 1824) (Anthobranchia) – New South Wales, Australia, G *Spurilla major *(Eliot, 1903) (Cladobranchia) – Queensland, Australia, H *Cuthona sibogae *Bergh, 1905 (Cladobranchia) – Queensland, Australia

Only in very few species of Opisthobranchia, the shell is big enough so that the animal can withdraw completely. In most species the shell is reduced in size, internalised or lost completely. Opisthobranchs are much less diverse in species numbers (5000 to 6000) than the terrestrial Stylommatophora (about 30000 species), or the shelled marine gastropods, in former times named "Prosobranchia" (60000 species), but they show many biological features that are unique or rare in the animal kingdom and that are often related to foraging or defensive strategies [2]. These include incorporation and usage of intact chloroplasts from algal cells for feeding strategies (Sacoglossa, Fig. 1D) [3], or storage of intact cnidocysts from cnidarians for defence (Aeolidoidea, Fig. 2E, G, H) [4]. Some of them are able to synthesize toxic compounds or to uptake these secondary metabolites from their food in order to use them as repellents (Nudibranchia, Chromodorididae, Fig. 2C) [5]. Many of these biological phenomena are hardly understood because investigation of biological data is scarce. Evolution of different strategies is not known, because of the lack of well-supported phylogenetic analyses.

Knowledge on the different subgroups of the Opisthobranchia differs according to their availability and spectacular appearance. For example, many more data on feeding strategies and other biological features are available for the beautifully coloured Nudibranchia (Fig. 2), than for the tiny and inconspicuous Acochlidacea. The authors have worked on different aspects of the biology of the Opisthobranchia for many years, trying to promote our understanding of this peculiar group. Although the Opisthobranchia is a rather small taxon, this group is ideal for evolutionary studies. Recently, Wägele reviewed potential key characters that might have enhanced radiation within the Opisthobranchia [2]. She has used a working hypothesis on opisthobranch phylogeny and published data on different strategies to deduce her proposals. She discussed the gizzard in Cephalaspidea, kleptoplasty in Sacoglossa, kleptocnides in Aeolidoidea, symbiosis with unicellular algae in *Phyllodesmium *Ehrenberg, 1831 and mantle dermal formations in Chromodorididae. Cimino and Ghiselin [6] and Cimino et al. [7] discussed the loss of the shell and the acquisition of toxic substances as a driving force in the evolution of Sacoglossa. Glandular structures and acquisition of chemical defence is subject of several reviews [8-13]. In the present review, our knowledge on Opisthobranchia is briefly summarized with emphasis on reduction of the shell and its implications on life history, especially regarding foraging and defence. Additionally new results on several glandular structures are presented. Some glands are described here for the first time. We point to a new aspect in the evolution of defensive devices. Their primary function as excretory or detoxification organs should be taken into consideration. It is beyond the scope of this review to include all new data on morphology, histology and phylogeny, as well as the literature published on the Opisthobranchia. The intention is to draw attention to a fascinating group of animals with a species number of manageable size, and in which similar evolutionary traits occurring in different groups at the same time and their implications can be analysed.

## Results

**Figure 3:**
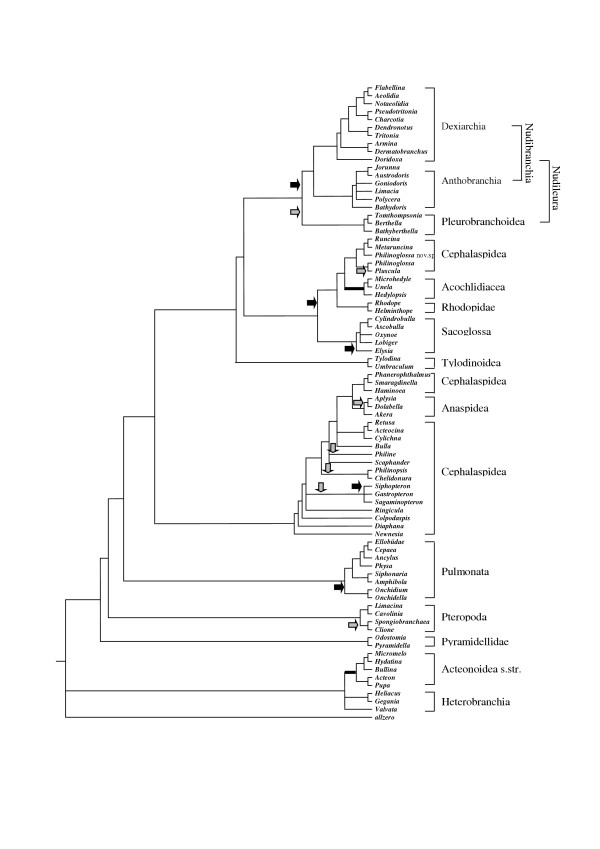
**Phylogeny of Opisthobranchia. **Cladogram based on morphological data. Grey arrows indicate internalisation, black arrows the loss, of the shell. Positions of Acteonoidea, Tylodinoidea and Acochlidacea are marked by bold lines, because they differ from those on the gene-based tree (Fig. 4)

**Figure 4:**
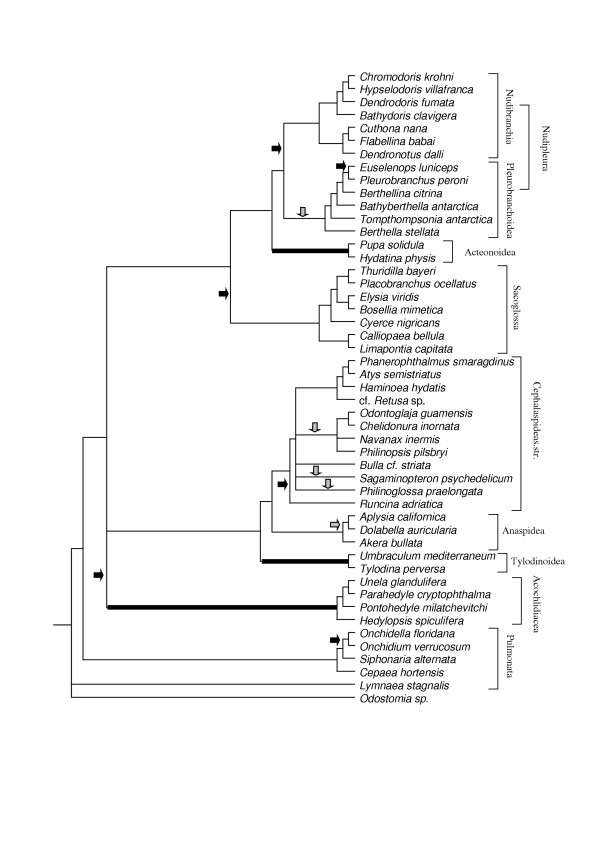
**Phylogeny of Opisthobranchia. **Cladogram based on 18S and 28S gene, after Vonnemann et al. (in press). Positions of Acteonoidea, Tylodinoidea and Acochlidacea are marked by bold lines, because they differ from those on the morphology-based tree (Fig. 3). Grey arrows indicate the internalisation, black arrows the loss, of the shell.

Figure 3 represents a preliminary phylogenetic tree of the Opisthobranchia, as well as a few members of the Pulmonata and basal Heterobranchia. This tree is based on data obtained by morphological and histological analyses. Characters are listed in table 1 (see additional file 2) and the data matrix in table 2 (see additional file 1). A complete discussion of characters and obtained trees is in preparation. In the tree presented here, all characters are treated as unweighted and unordered (see methods below). A similar comprehensive tree of the Opisthobranchia, based on 18S and 28S genes is published by Vonnemann et al. [14]. These authors did not include basal Heterobranchia, Pteropoda and enigmatic forms, e.g., the Rhodopidae (Fig. 4). Comparison of these two most recently obtained morphological and genetic trees shows that nearly all major opisthobranch subgroups are monophyletic, but the position of some of these groups differs between the cladograms. These differences mainly concern the position of the Acteonoidea, Tylodinoidea and Acochlidacea (highlighted in both Figs. 3 and 4 by bold bars). Nevertheless evolutionary traits concerning the fate of the shell can be detected within well-defined clades (see Figs. 3 and 4: shell internalisation is indicated by grey arrows, shell loss by black arrows). Several groups included in the trees will not be considered in this study, because they are not assigned to the Opisthobranchia. These groups are the basal Heterobranchia, Pyramidellidae and Pulmonata. Furthermore, this discussion focuses more closely on the morphology-based tree, because more taxa are included there.

### Description of monophyletic groups

#### Acteonoidea

**Figure 5:**
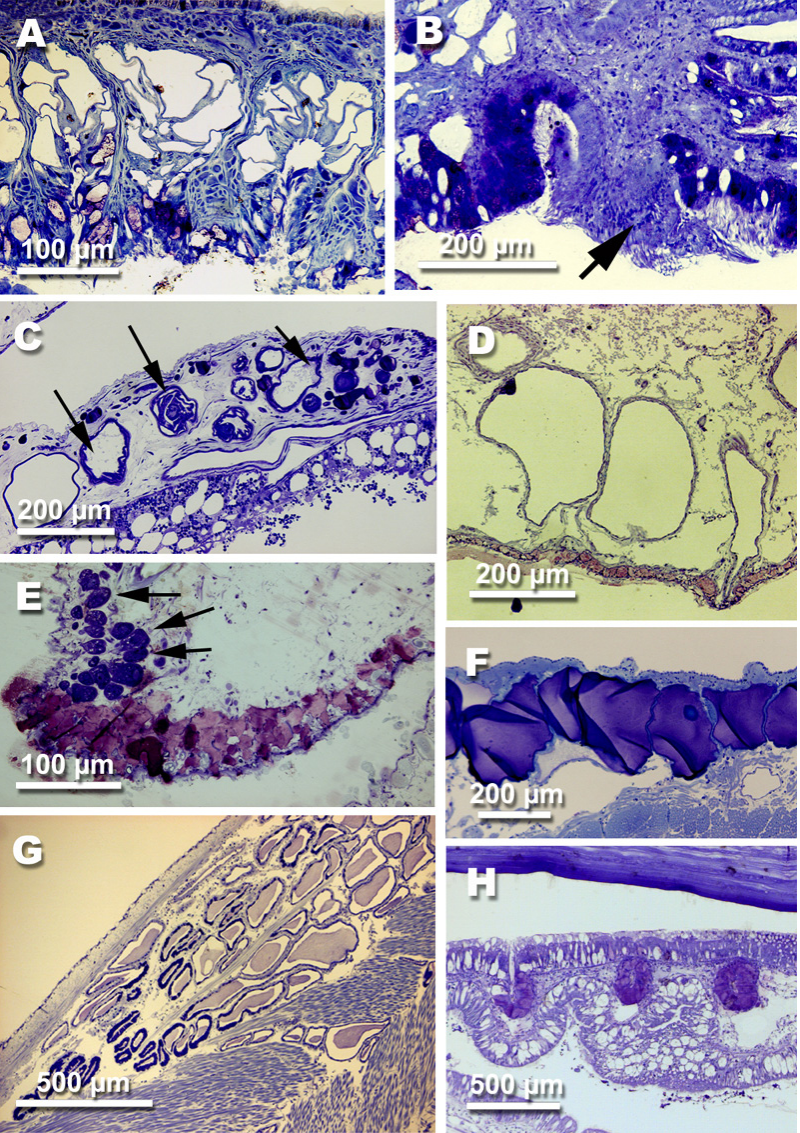
**Glandular structures in opisthobranch species. **A *Acteon tornatilis *(Linné, 1758) (Acteonoidea), mantle rim glands. B *Bullina lineata *(Gray, 1825) (Acteonoidea), hypobranchial gland. Note the ciliated raphe (arrow). C *Clione limacina *(Phipps, 1774) (Pteropoda, Gymnosomata), large single cellular glands (arrows). D *Haminoea antillarum *(d'Orbigny, 1841) (Cephalaspidea), Blochmann glands. E *Chelidonura tsurugensis *Baba & Abe, 1959 (Cephalaspidea), hypobranchial gland with violet stained glandular cells and above (arrow), single bluish stained glandular cells. F *Dolabrifera dolabrifera *(Cuvier, 1817) (Anaspidea), gland of Bohadsch, or opaline gland. G *Umbraculum umbraculum *(Lightfoot, 1786) (Tylodinoidea), dorsal mantle gland. H *Tylodina perversa *(Gmelin, 1791) (Tylodinoidea), dorsal mantle glands in the free mantle rim; above lies the shell.

The families Acteonidae and Hydatinidae form sister-groups, the position of the debatable monophylum is under discussion. All acteonoids have a shell that resembles that of many prosobranchs (Fig. 1A). Some of the members are able to withdraw completely into the shell and to close the shell with an operculum, e.g. *Acteon tornatilis *(Linné, 1758). Acteonidae and Hydatinidae are carnivorous and mainly feed on polychaetes. No defensive strategies are known from these animals although histological investigations show a highly glandular area in the mantle cavity and the mantle rim. The mantle rim glands, for example, are very conspicuous. These comprise large epithelial cells that are filled with a non-staining vacuole (Fig. 5A). The glandular area is highly folded. The cells appear to lie subepithelially due to their size. They alternate with small ciliated cells. The hypobranchial gland in the roof of the mantle cavity is small and consists of violet-staining epithelial cells indicating acid mucopolysaccharides (Fig. 5B).

#### Pteropoda

This group living exclusively in pelagic waters comprises two major clades, the herbivorous Thecosomata and the carnivorous Gymnosomata. Many thecosomates still have a shell whereas the Gymnosomata have lost it. The latter feed on the former. Many morphological adaptations have occurred due to their life in pelagic waters. Defensive mechanisms are hardly known from pteropods. Whereas the Thecosomata do not show specialized defensive glandular structures in the outer epithelium, peculiar structures of rather unknown function can be found in the two species of Gymnosomata investigated here. Both species show single large glandular cells, with one vacuole and a larger nucleus. The contents of the vacuoles only stain in smaller, probably immature, cells. In larger cells they are translucent (Fig. 5C).

#### Cephalaspidea

**Figure 6:**
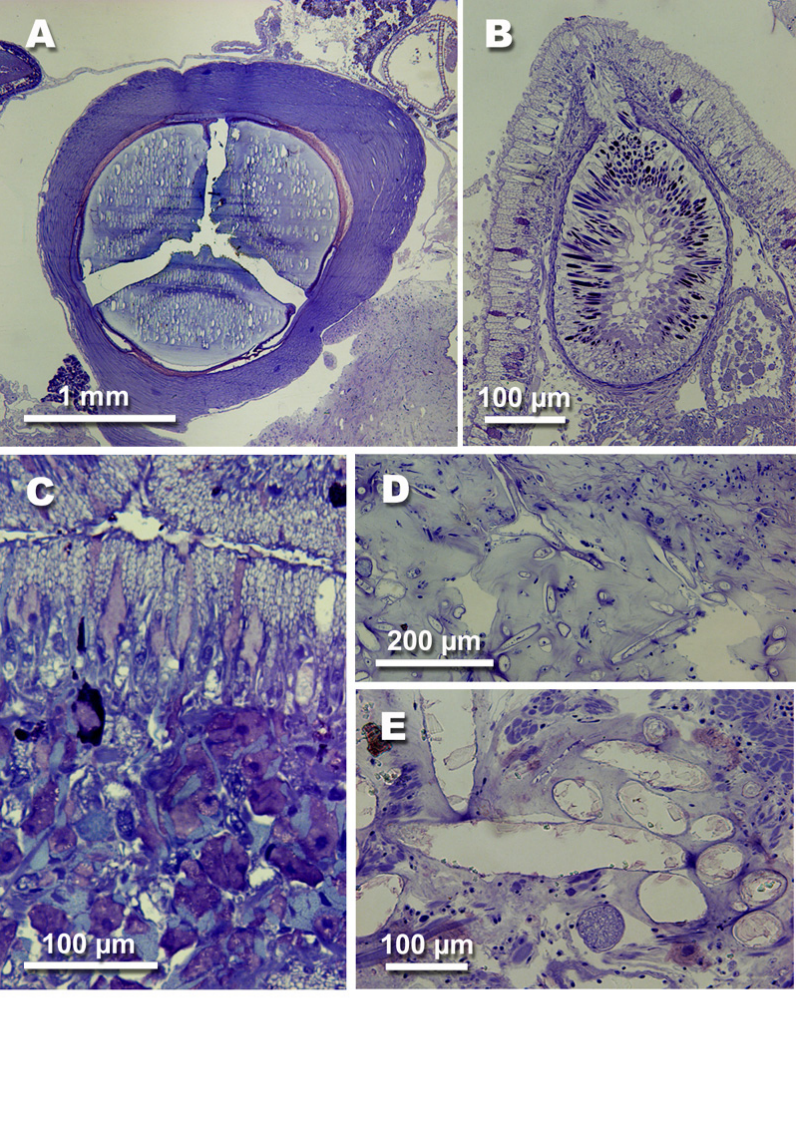
**Anatomical features characteristic for some opisthobranch species. **A *Haminoea antillarum *(d'Orbigny, 1841) (Cephalaspidea), gizzard. B *Aeolidia papillosa *(Linné, 1761) (Nudibranchia, Dexiarchia, Aeolidoidea), longitudinal section of ceras with cnidosac with cleptocnides and outleading duct. C *Aeolidia papillosa*, oral tube with subepithelial glandular tissue. D *Tomthompsonia antarctica *(Thiele, J., 1912) (Pleurobranchoidea), spicules in notal tissue. E *Onchidoris bilamellata *(Linnaeus, 1767) (Nudibranchia, Anthobranchia), spicules in notal tissue.

Members of following families have been included in this analysis: Smaragdinellidae, Haminoeidae, Retusidae, Cylichnidae, Bullidae, Philinidae, Aglajidae, Gastropteridae and Diaphanidae. Monophyly on family level was not recovered for the Diaphanidae and Cylichnidae. Furthermore, the Cephalaspidea (Acteonoidea excluded) is not monophyletic due to the inclusion of the Anaspidea. In our analysis, the gizzard-bearing groups form one clade, representing the Anaspidea (Fig. 1E) and the Cephalaspidean families Smaragdinellidae, Haminoeidae, Retusidae, Cylichnidae (Fig. 1B), Bullidae and Philinidae. *Runcina *Forbes & Hanley, 1853 and a yet undescribed *Philinoglossa *Hertling, 1932, both with a gizzard, are not part of that monophyletic group. The gizzard is a muscular oesophagus with 3 to 10 large gizzard plates that function like a grinding mill (Fig. 6A). Feeding strategies are highly diverse within these different groups. Herbivory is known only from cephalaspids with gizzard plates, whereas carnivory is widely spread within all other cephalaspidean groups with or without a gizzard. Prey items are mainly polychaetes, bivalves and in a few cases congeners. *Runcina *and *Philinoglossa *feed on diatoms.

Whereas many herbivorous slugs still have an external shell, e.g. *Bulla *Linné, 1758, *Haminoea *Turton & Kingston, 1830, *Retusa *Brown, 1827, *Cylichna *Lovén, 1846, etc., only a few carnivorous cephalaspids have retained a large shell (e.g. *Scaphander *Montfort, 1810) (Fig. 1B). Many have an internalized shell (Fig. 1C, *Chelidonura *Adams, 1850, *Gastropteron *Meckel in Kosse, 1813) or have lost it all together (*Siphopteron *Gosliner, 1989). Cephalaspideans have several glandular structures, although their function is hardly understood. The hypobranchial gland is composed of epithelial cells staining violet (Fig. 5E). This gland can be very voluminous (e.g. in *Haminoea callidegenita *Gibson & Chia, 1989) or can be reduced (in many cephalaspids with small and reduced mantle cavity, e.g. *Chelidonura tsurugensis *Baba & Abe, 1959). In many Cephalaspidea single glandular cells can be observed that stain bluish and open to the outside by a small duct (Fig. 5E, glandular cells above hypobranchial gland). These glands usually are confined to the mantle cavity roof. A special type of single gland is present in very few members of the Cephalaspidea, namely the Blochmann's glands. One characteristic is the duct leading to the outside that is composed of a few small cuboidal cells. The contents of these glands do not stain (Fig. 5D).

#### Anaspidea

This group, which is mainly characterized by two pairs of head tentacles (Fig. 1E), is closely related to the gizzard bearing cephalaspids. Nearly all species have a reduced shell or no shell at all (none of the latter species are included in the analyses presented here). In general, Anaspidea feed on red, brown and green algae. The group is known for their defensive habits, by using an ink gland when disturbed. The glands are typical Blochmann's glands already described for Cephalaspidea. Another gland is also widespread in Anaspidea and assumed to be an additional defensive gland, the so-called gland of Bohadsch, or opaline gland (Fig. 5F). It is composed of large cells containing a large nucleus. In general they are considered to be special forms of the Blochmann's gland [8,15]. The glands open at the bottom of the visceral cavity and stain violet. In a few species single glandular cells are arranged around a single opening. Additionally, the single blue-stained subepithelial glandular cells already described in Cephalaspidea (see Fig. 5E) are present in the dorsal mantle cavity.

#### Tylodinoidea

This tiny group is characterized by a rather large foot, and the umbrella-like shell covering the viscera, but not the foot (Fig. 1F). Data on biology of this small group are scarce. Probably all of them feed on poriferans. The Mediterranean *Tylodina perversa *(Gmelin, 1791) fosters the secondary metabolites from its exclusive food, the sponge *Aplysina aerophoba *Schmidt, 1862 [16]. Becerro et al. [17] demonstrated that the slug actively selects for sponges with a high concentration of cyanobacteria, whereas sponges without these bacteria (e.g. *A. aerophoba *from deeper waters, or *A. cavernicola*) were neglected. Histological investigation show that the Tylodinoidea have several peculiar glands. In the dorsal mantle tissue of *Umbraculum umbraculum *(Lightfoot, 1786), many tubules of a highly ramifying gland (Fig. 5G) lead to one or two main ducts that open to the outside in the anterior mantle rim above the mouth. *Tylodina perversa *has glandular tissue in the same area as *U. umbraculum*, but it has several ducts leading to the outside, all lying at the anterior dorsal mantle rim (Fig. 5H).

#### Sacoglossa

**Figure 7:**
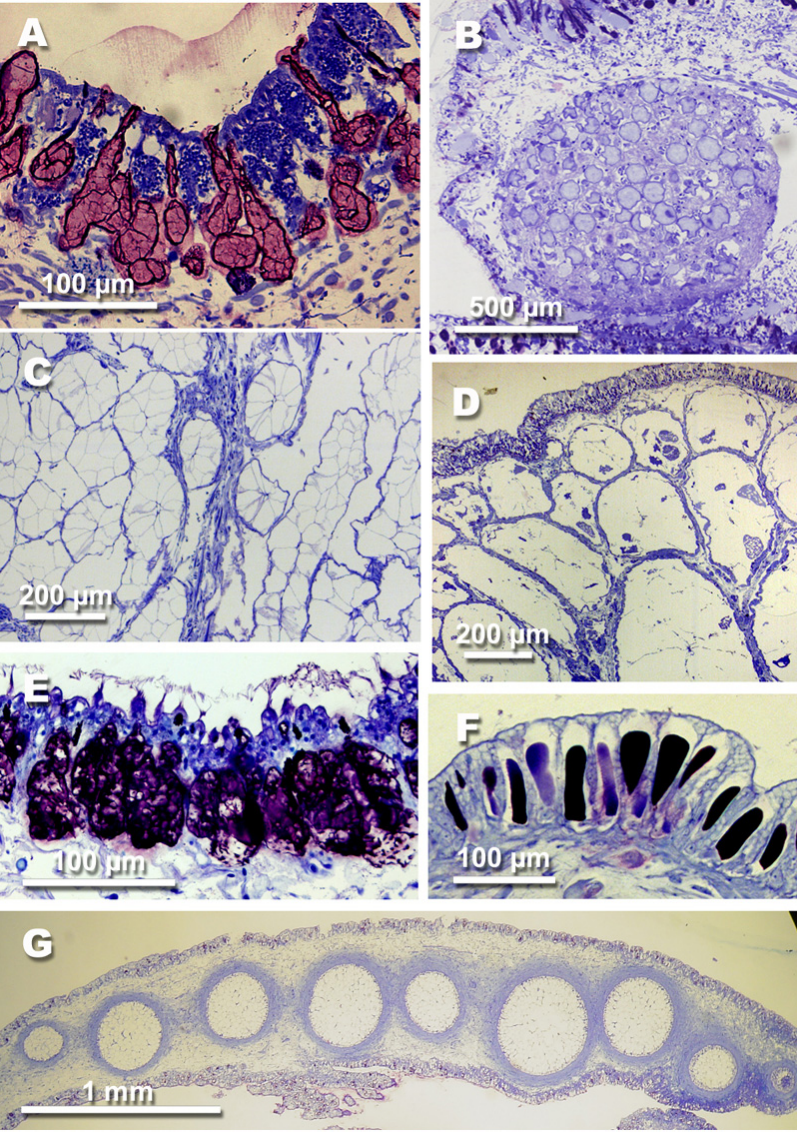
**Glandular structures in opisthobranch species. **A *Elysia crispata *(Moerch, 1863) (Sacoglossa), subepithelial glandular cells in dorsal epithelium. B *Plakobranchus ocellatus *van Hasselt, 1824 (Sacoglossa), mantle dermal formation in the edge of the parapodia. C *Berthellina citrina *(Rüppell & Leuckart, 1828) (Pleurobranchoidea), median buccal gland in visceral cavity, producing sulphuric acid. D *Berthella edwardsi *(Vayssiére, 1896) (Pleurobranchoidea), acid glands lying in the notum tissue. E *Thecacera pennigera *(Montagu, 1815) (Nudibranchia, Anthobranchia), subepithelial glandular cells in dorsal epithelium. F *Marionia blainvillea *(Risso, 1818) (Nudibranchia, Dexiarchia, Dendronotoidea), epithelial glandular cells with unusual large vacuoles filled with homogenously stained contents. G *Risbecia tryoni *(Garrett, 1873) (Nudibranchia, Anthobranchia), mantle dermal formations (MDFs) along the posterior mantle rim.

This group is monophyletic. In the morphological tree presented here (Fig. 3), the non-shelled *Elysia *species appears as the most basal one, whereas shell-bearing sacoglossans, like *Cylindrobulla*, are more derived. External morphology of sacoglossan species shows a high diversity. A rather primitive large and coiled shell is present in *Cylindrobulla *Fischer, 1857 and *Ascobulla *Marcus Ev., 1972. Others, like *Oxynoe *Rafinesque, 1814 and *Lobiger *Krohn, 1847, have tiny shells, whereas the shell is lost in all Elysiidae (Fig. 1D). A peculiar bivalved shell is present in the family Juliidae. Many sacoglossans feed on green algae (Ulvophycea) by piercing the algal cells with their radular teeth and by sucking the contents into their digestive tract. Defensive glands are not so obvious, but cryptic appearance obtained by an uptake and storage of chloroplasts is evident for many species as can be seen for *Elysiella pusilla *Bergh, 1872 feeding on the alga *Halimeda *Lamouroux, 1816 (Fig. 1D). Investigated Sacoglossa are characterized by many subepithelial glands with violet-stained contents (Fig. 7A). But the quantity of these cells differs to a great extent among species. *Placobranchus ocellatus *van Hasselt, 1824, a shell-less slug, is unique in having many globular structures arranged along the edge of the mantle rim (Fig. 7B). These structures have a diameter of nearly 1 mm and are composed of many cells each with a large vacuole. The contents of the vacuole stain bluish.

#### Pleurobranchoidea

Shells, when present, are internalised (Fig. 1G). Pleurobranch species feed on different prey items, but they are all carnivorous, some even feed on congeners. Typical for the group is a huge acid gland lying in the visceral cavity and opening into the oral tube next to the mouth. The gland is composed of huge cells with non-staining vacuoles (Fig. 7C). Additionally, several species show a highly glandular notum epithelium with huge subepithelial glandular follicles composed of cells with non-staining vacuoles (Fig. 7D). Several members, like *Tomthompsonia *Wägele & Hain, 1991, have spicules in their notum (Fig. 6D).

#### Nudibranchia

All members of the monophyletic Nudibranchia have lost the shell completely (Fig. 2A–H). This taxon, with about 3000 species and a high diversity in shape and in biology, is the largest opisthobranch group and comprises about half of the known opisthobranch species. Two monophyletic clades can be recognized, the Anthobranchia (Fig. 2B, C, F) and the Dexiarchia (Fig. 2A, D, E, G, H). The former mainly feed on Porifera, Bryozoa and Tunicata, the latter on Cnidaria, mainly on octocorals. Defensive strategies are very diverse in the Nudibranchia and comprise different techniques. Many species of the Anthobranchia, especially those feeding on sponges, have spicules in the notum (Fig. 6E). Many species are characterized by a highly glandular epidermis (Fig. 7E). Nearly all members of the very species-rich family Chromodorididae (Fig. 2B) have so called mantle dermal formations (MDFs) lying in the mantle tissue (Fig. 7G). These are globular structures with a diameter of up to 1 mm. MDFs are composed of cells with huge non-staining vacuoles.

Many Dexiarchia species are also characterized by a glandular epidermis. Especially members of the Dendronotoidea (here *Tritonia *Cuvier, 1798 and *Dendronotus *Alder & Hancock, 1845) are characterized by epithelial glandular cells in which the vacuole is filled with homogenously stained contents (Fig. 7F). Aeolidoidea have so called cnidosacs at the tip of their notal cerata that represent the apical parts of the digestive glandular tubes running within the cerata. In these cnidosacs, the cnidocysts from their cnidarian prey are stored and can be used against potential enemies (Fig. 6B).

### Other groups in the cladogram

According to the phylogeny presented in Fig. 3, several taxa are united in a monophylum. Systematic relationships of some groups have been discussed for a long time (Acochlidiacea, Rhodopidae), others have been considered to belong to the Cephalaspidea (Philinoglossidae, Runcinidae). Their sister-taxon relationship is not solved yet and the presented cladogram is debatable. Nevertheless they have in common some evolutionary traits, e.g. the complete loss of the shell, their small size compared to other opisthobranchs and their food preference for diatoms and detritus. According to histological results, no particular defensive glands could be detected and defensive strategies probably lay in habits. Many of them burrow in sand and/or are cryptic in colour.

## Discussion

In accordance with published phylogenies based on morphology [18-20], or based on genes [14,21,22] all major clades presented here are monophyletic (Acteonoidea – but see the study by Mikkelsen [23], Cephalaspidea with Anaspidea included, Sacoglossa, Tylodinoidea, Pleurobranchoidea, Nudibranchia, Anthobranchia, Cladobranchia). Concerning relationships of major groups, several congruencies with former analyses can be observed: The sister-taxon relationship of the Nudibranchia and Pleurobranchoidea [14,24] is found in nearly all analyses. This group nowadays is called Nudipleura Wägele & Willan, 2000. A further consistent grouping is formed by Cephalaspidea s.str. and Anaspidea. This relationship was already discussed by Mikkelsen [20,23]. All other presented groupings are still under debate. In our morphology-based tree, *Elysia *represents the most basal taxon within the Sacoglossa. This contradicts other available phylogenetic analyses and has to be considered with care. Jensen presented a thorough phylogenetic analysis on Sacoglossa [25]. According to her results, taxa with a shell are more basal and shell reduction occurred at least twice within the Sacoglossa. In the discussion below, we follow the results of Jensen and Mikkelsen and consider the shell-bearing sacoglossans as more basal [23,25].

Despite these incongruities, a discussion of shell reduction in the different groups and its implications on life history (habitat, feeding and defensive strategies) can be undertaken, and will serve as a guideline for further investigations.

### Implications on life style

A shell is generally considered to be a protection against predators, such as fish, crabs and other vagile organisms. "If the shell of a whelk (e.g. *Buccinum*, a "prosobranchiate" caenogastropod, annotation of the authors) is broken away and the soft animal is then offered to a hungry cod, it is eaten readily." (p: 115) [26]. Reduction, internalisation or loss of the shell within Opisthobranchia implies other defensive strategies. Shell reduction within molluscs is uncommon, and occurs mainly in the highly mobile cephalopods. In gastropods, shell loss is rare in paraphyletic prosobranchs, and known only from few groups of Pulmonata, e.g., the Gymnomorpha and the stylommatophoran groups Arionidae and Limacidae. However, shell reduction occurred many times within the different subgroups of Opisthobranchia. Here, an internalization or complete loss occurs within the Cephalaspidea s.str, Anaspidea, Sacoglossa, Acochlidiacea and Pleurobranchoidea (Fig. 3). Whereas complete loss of the shell is not known from any member of the small taxon Tylodinoidea (about 15 species), this character state occurs in the stemline of the Nudibranchia and Gymnosomata. When estimating species numbers with no shell or a rather tiny internal shell and comparing this to the number of species with a larger external shell, the former outnumber the latter by far.

**Figure 8:**
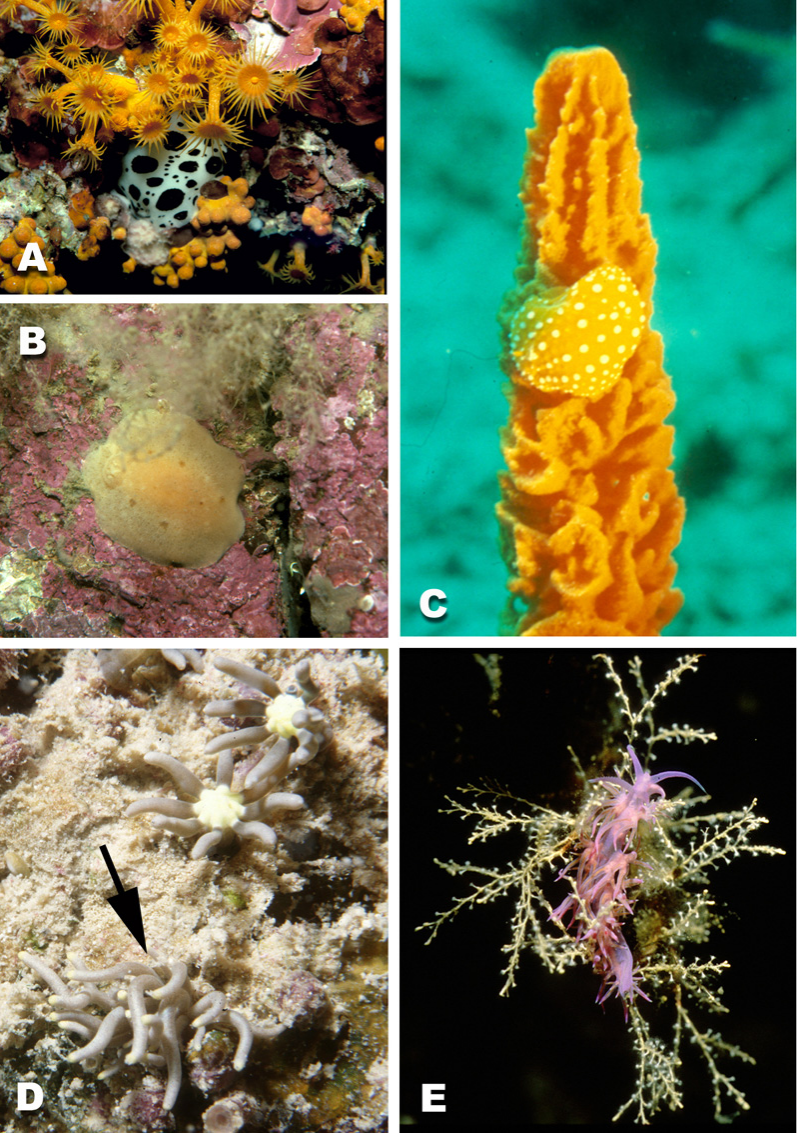
**Examples of cryptic nudibranch species. **A *Discodoris atromaculata *Bergh, 1905 (Anthobranchia) from the Mediterranean, attached to the roof of a cave between *Parazoanthus*, B *Jorunna tomentosa *(Cuvier, 1804) (Anthobranchia) from the Northern Sea, attached to rocks with corralineacean red algae and mimicking a sponge (Halichondria), C *Phyllidia flava *Aradas, 1847 (Anthobranchia) from the Mediterranean Sea, feeding on *Axinella *cf. *cannabina *and incorporating the dyes. D *Phyllodesmium briareum *(Bergh, 1896) (arrow, Dexiarchia, Aeolidoidea) from the Indo Pacific, mimicking its food, the soft coral *Briareum violacea*. E *Flabellina affinis *(Gmelin, 1791) (Dexiarchia, Aeolidoidea) from the Mediterranean, crawling on its food *Eudendrium racemosus *(Cnidaria, Hydrozoa).

Loss of the shell therefore can be assumed to have advantages compared to the presence of a protective but heavy shell. Advantages probably lay in the exploration of new habitats, which are more difficult to reach when being protected by a shell. This can be observed e.g. in a subgroup of the Cladobranchia. The Aeolidoidea are able to graze on fragile hydrozoans (Fig. 8E). This kind of prey is used by few other invertebrates, e.g., Solenogastres, members of the Pycnogonida and of the Amphipoda [27-29]. Burrowing forms with a shell, e.g., *Scaphander *or *Acteon *Montfort, 1810, have an elaborate cephalic shield that partially covers the shell and renders them streamlined. Loss of the shell probably enables slugs to search for food in sandy or muddy habitats more easily. This is the case for members of the Cephalaspidea s.str. and Acochlidiacea.

Basal members of the Sacoglossa have retained a shell, but more derived ones have lost it. Shell loss allowed evolution of a phenomenon that is unique in the animal kingdom. Sacoglossa in general feed on algae by piercing the cells with their tooth and sucking out the contents of the cell. The cytoplasm is digested, but in many species (e.g. *Elysia timida *(Risso, 1818), *Placobranchus ocellatus*) the chloroplasts are stored in distinct branches of the digestive gland. Here they are stored for a period of several days to months [30]. For this phenomenon, the term "cleptoplasty" is used by several authors [25]. The functioning chloroplasts continue with photosynthesis within the slug and provide nutritional metabolites for the metabolism of the gastropod [3,13,31,32]. Penetration of light into the slug would be hindered by the possession of a shell. A similar system is observed in members of the Nudibranchia, e.g., in *Phyllodesmium jakobsenae *Burghardt & Wägele, 2004, or *Melibe bucephala *Bergh, 1902) [33,34]. Here unicellular algae (zooxanthellae) from the coral food or from the free water column are stored in the digestive system and metabolites of these zooxanthellae are used for the slug's own purposes [35-37]. According to published phylogenies and to our own results (unpublished data of both authors) on Sacoglossa and Nudibranchia, it can be assumed that uptake of chloroplasts or zooxanthellae first enhanced crypsis (Fig. 1D) [25]. The short-term storage allows a continuation of the photosynthetic activity of chloroplasts within the slug. Storage over a longer period allowed the reduction of food uptake with the possibilities to search for new and/or less frequent prey organisms [2,38]. The most effective symbiotic relationships are known for the sacoglossan *Elysia chlorotica *Gould, 1870, which can survive eight months without food [3], the aeolid *Pteraeolidia ianthina *(Angas, 1864) and the dendronotoidean *Melibe bucephala*, both of which survived in our aquaria for 10 months without food (Burghardt & Wägele unpublished data).

### Implications on defence

Loss of a shell as a protective structure led to an array of different defensive structures. Some of these traits can be observed as a combination in one and the same species.

Crypsis can be observed in many groups and is very often achieved by incorporation of the same dyes from the food (Fig. 8C, *Phyllidia flava *Aradas, 1847). Cryptic appearance also is achieved by mimicking the same patterns or even outline of the substrate. *Corambe pacifica *MacFarland & O'Donoghue, 1929 perfectly mimics the colour patterns of its prey, the bryozoan *Membranipora *de Blainville, 1830. *Phyllodesmium jakobsenae *mimics the feathered polyps of the soft coral *Xenia *Lamarck, 1816, on which it lives [33], whereas the cerata of *P. briareum *(Bergh, 1896) are smooth like the tentacles of its prey, the soft coral *Briareum *Blainville, 1830 (Fig. 8D). Zebra effects are achieved by patterns with blotches like that in *Peltodoris atromaculata *Bergh, 1880 (Fig. 8A) or by stripes. Looking like unpalatable sponges (Fig. 8B, *Jorunna tomentosa *(Cuvier, 1804)) is very common in spicule-bearing dorids. According to Gosliner [39], the cryptic species are rather basal taxa, whereas the taxa with aposematic colour patterns are more derived – a hypothesis that has yet to be proven by thorough phylogenetic analyses that include all species of the subgroup in question.

A unique defensive strategy within animals is the storage of cnidocysts ("cleptocnides"), which is typical for nearly all members of the cladobranch Aeolidoidea [4,9,40]. This group mainly feeds on cnidarians, with priority on Hydrozoa. The mechanisms of the uptake of cnidocysts, so that explosion is not triggered during consumption, are still not understood. It is assumed that the slug exudes a mucus to hinder explosion [9,26]. Investigated aeolids, like *Aeolidia papillosa *(Linné, 1761), have a highly glandular oral tube (Fig. 6C) that supports this hypothesis. Another theory implies that there occurs a kind of acclimation process, similar to that discussed between sea anemones and anemone fish [41]. According to the investigations of Greenwood and Mariscal [42] only immature cnidocysts are stored in the cnidosac, whereas mature ones are digested. But, histological investigation of many aeolids directly collected from their food have not revealed high numbers of exploded cnidocysts in the stomach (unpublished data of HW). Only *Notaeolidia schmekelae *Wägele, 1990 from the Antarctic Ocean has been observed to have many exploded cnidocysts in its digestive tract [43].

Presence of spicules in the notum as a defensive strategy was discussed by several authors [10,44]. Spicules are present in many shell-less Anthobranchia and Acochlidiacea, but also in members of the Pleurobranchoidea, which sometimes have an internalised small shell. Spicules never occur in opisthobranchs with a larger shell. Cattaneo-Vietti et al. investigated the mineral composition of dorid spicules and found calcite (CaCO_3_) and brucite (Mg(OH)_2_) [45]. Smaller spherules are composed only of calcite. Harris described feeding experiments offering various opisthobranchs to specimens of *Navanax *Pilsbry, 1895 (Cephalaspidea), who is a ferocious predator on opisthobranchs [10]. This species rejected all spiculose dorids.

Another evolutionary trait for defence, and discussed as a prerequisite for shell reduction at least in sacoglossans [13], is the uptake or *de novo *synthesis of secondary metabolites that are toxic to possible predators [5,46]. Uptake by feeding on toxic prey (mainly algae, Porifera, Bryozoa, Tunicata and Cnidaria) is the major source of compounds, whereas *de novo *synthesis is known only from few taxa [5]. When dietary derived, Avila called these cleptochemicals, following the terms cleptoplasts and cleptocnides for incorporation and use of chloroplasts in Sacoglossa and cnidocysts in Aeolidoidea [5]. Literature on chemical compounds in opisthobranchs is numerous. Some reviews summarize our knowledge [5,7,46-49]. Compounds mainly belong to the terpenoids, especially the insoluble sesquiterpenoids and diterpenoids. Little is known about the function of the biological compounds, although their defensive tasks are very often postulated [5-7,46]. Few feeding experiments have been performed in the past, demonstrating a toxic effect on crustaceans and/or fish [26,50]. Also the translocation from prey into the slug, and the transformation by changing the chemical structures either by degradation through digestion, or by an active mechanism into a more effective chemical, is hardly understood [5,7]. Location of the compounds is investigated only for few species, by analysing certain parts of the body [51], or even by isolating larger organs, like the MDFs [52]. Tracing the compounds within the tissue, or even cells, using immunohistochemical methods has never been done. Therefore, it is not possible to correlate chemical bioactivity with certain histological structures, except for the mantle dermal formations in the species *Hypselodoris webbi *(Chromodorididae) [52].

Inorganic compounds, like sulphuric acid are produced in few groups. Their function and location is better known due to the extensive work of Thompson [53-55]. He analysed the production of sulphuric acid in different members of Gastropoda, including members of the Pleurobranchoidea, Cephalaspidea and Dorididae. The exudated acid contains inorganic chloride and sulphate anions, and traces of organic substances. He was able to localize the acid by histochemistry within the large vacuoles in the median buccal gland (Fig. 7C) and the subepithelial glands of Pleurobranchoidea (Fig. 7D). There, the acid is held in active form [53]. Gillete et al. investigated the role of the central nervous system and peripheral nerves for exudation and showed positive feed back [56].

Broad histological investigations of the Opisthobranchia show that many species are characterized by a large array of glandular structures [8,11,57-59]. These comprise single glandular cells lying in the outer epithelia, or subepithelially. Glandular follicles composed of several cells usually lie subepidermally and open via a duct to the outside. Larger organs are the MDFs, or the glandular tubules of the median buccal gland in the Pleurobranchoidea. Some of these structures have been known for a long time and their defensive tasks were discussed in more detail by Hoffmann [8]. Well known are the ink gland (Blochmann's glands) and the opaline gland (Bohadsch gland) in the Anaspidea. Both glands exude substances that have been shown to be toxic to cnidarians [1]. Probably these substances also caused severe damage of the liver of a 40-year-old man, who ate *Aplysia kurodai *Baba, 1937 [60]. By experimental studies it was shown that the repellent substance in the ink gland is a monomethyl ester of phycoerythrobilin and is derived from phycoerythrin from the consumed red algae [61]. The role of the opaline gland is less known. According to Carté, the prosteroglandine with the highest known biological activity is Dolastatin 10, a natural product extracted from the anaspidean *Dolabella auricularia *(Lightfoot, 1786) [62]. This large species of more than 10 cm lives on the intertidal flats in the tropical Indo-Pacific, where it would represent an ideal food for birds and fishes, if not for that highly toxic chemical. This substance is already applied in medical treatments (see ), and seems to be one of the most potent anticancer agents.

Information on other glandular structures are rare, and nearly nothing is known about their contents and their functions. At the moment we are not able to trace the different substances in these glands to find out whether there are any constraints concerning structure (and therefore function) and the stored chemicals.

Only few hypotheses are formulated concerning acquisition of toxicity and loss of the shell. Faulkner & Ghiselin assumed that chemical defence based on metabolites derived from food preceded the reduction of the shell and that chemical defence has been a driving force behind the evolution of Opisthobranchia [13,46,63]. Cimino et al., by analysing the different compounds and their origin, came to the conclusion that evolution within the Sacoglossa started with the uptake and storage of sesquiterpenoids from algae in species still having a shell [7]. Within the shell-less members of the family Elysiidae, diterpenoids from the algae were stored, whereas in highly evolved forms, like *Elysia timida*, the slugs switched to a *de novo *synthesis of polyproprionates. Cimino & Ghiselin also mentioned that handling and utilization of a particular kind of defensive metabolite allowed the switch to food with similar compounds quite easily, and therefore has driven adaptive radiation [46]. As an example they named the dorids and in particular the family Chromodorididae, which show a large array of usage of biochemicals from different sponges. Again, the bio-synthesis of compounds, as observed in *Dendrodoris *Ehrenberg, 1831, is considered to be the most derived form of defence within the Anthobranchia.

Information on defensive strategies, as listed above, is available now for several groups of the Opisthobranchia. More and more reliable phylogenies are becoming available, which allow the identification of well-supported branches and stemlines. Combining this knowledge, it becomes evident that several defensive systems evolved before the loss of the shell (several glandular structures, e. g., the hypobranchial gland, mantle rim glands, Bohadsch gland). Here we would like to extend the hypothesis of Cimino et al. by addressing the problem of excretion [7]. It can not be ruled out that certain glandular structures evolved as a kind of excretory system to get rid of ingested toxic substances. Therefore it is not storage in special organs that preceded the use of toxic substances, but the necessity to expel them. By analysing phylogeny, it is evident that many defensive structures evolved after the internalisation or loss of shells (e.g. acid glands in the notum, cleptocnides, MDFs).

But we still have to identify the location of the compounds for a better understanding of the evolutionary history concerning the acquisition of toxicity, which certainly was a driving force in the evolution of these fascinating opisthobranchs. New techniques, e.g., the oligonucleotide aptameres, could help to solve this question [64]. We also have to keep in mind that chemical substances might not only play a role in defence (allomones), but also in reproduction and development (pheromones).

## Conclusions

In this review it is shown that shell loss led to the evolution of a wide array of defensive strategies in Opisthobranchia. Nevertheless, it is not ruled out that some defensive mechanisms have evolved prior to complete loss of shell. This is evident when analysing the Acteonoidea. Members of this taxon have a rather thick and large shell, but also different glandular structures along the mantle fold, as well as in the mantle cavity. One working hypothesis for future research is that defensive glands evolved from simple storage organs while feeding on toxic prey. Evolution of special structures where toxic substances could be stored without further effect on the body and that functioned as a kind of excretory system could have been the prerequisite for employing these structures as defensive devices. To solve this question, thorough phylogenetic analyses are needed. Tracing toxic substances from the food into the different organs or glands by histochemistry or new analytical methods like aptameres will help to understand the tasks of these glands, and their role as excretory or as defensive organs, or as pheromone-producing organs involved in reproduction and development. Additional field and laboratory experiments with potential predators from the natural surroundings are necessary to understand the functioning of chemicals in the slugs.

## Methods

### Material collection

About 300 different species of Opisthobranchia have been collected and investigated by the authors in the past 20 years. Collecting was performed from the intertidal (e.g. Australia, Helgoland), to the sublitoral zones (e.g. Mediterranean Sea, North Atlantic, tropical waters in the Red Sea, Australia and others) down to depth of 1000 m (Antarctica). Collecting techniques comprised hand collecting in the intertidal and while diving, or using trawls, like the Agassiz trawl in Polar Seas. Specimens were preserved in 4 – 10 % formaldehyde/seawater for histological investigations, or 96% ethanol for molecular investigations.

### Investigation techniques

Investigation of morphology and anatomy was performed by macroscopical and histological techniques. For histological investigations, entire animals (when small in size) or parts of the animals were embedded in hydroxyethylmethacrylate for serial sectioning (2 μm). Sections were stained with toluidine blue and investigated by light-microscopy. Toluidine blue stains acid mucopolysaccharides in various shades of red to violet, whereas neutral mucopolysaccharides are staining in blue colours. Observation of living animals aided the understanding of external characters and life strategies. Data used for the phylogeny comprise 79 taxa and 110 characters based on morphology and histology. Polarity of characters was obtained by outgroup comparison with *Littorina littorea*. Due to some trivial characters, an all-zero outgroup was chosen. The characters are explained in full detail by Wägele & Klussmann-Kolb (in prep). Analyses were performed by PAUP 4.0 beta 3a (Swofford, 1999) [65]. Parameters of maximum parsimony analyses were: ACCTRAN, all characters unordered and unweighted; heuristic search options: stepwise addition = random, branch-swapping option = TBR.

## Authors' contributions

HW and AKK together carried out the phylogenetic analysis based on morphological and histological characters published here for the first time. HW drafted the manuscript. AKK helped to draft the manuscript. Both authors read and approved the final manuscript.

## Supplementary Material

Additional File 1 **Data matrix of characters **– 79 taxa and 110 characters are included. N = non applicable. This implies that the character is not present and can therefore not be coded. ? = character state not known. Genus with an asterisk indicate that information on this genus is extracted from literature. Detailed information will be given elsewhere in Wägele & Klussmann-Kolb (in prep.). Click here for file

Additional File 2 **Characters **– Characters and coding of character states used for phylogenetic analysis presented in Figure 3. Click here for file
