# Supplementary material for: Opisthobranchia (Mollusca, Gastropoda) – more than just slimy slugs. Shell reduction and its implications on defence and foraging
Source: Front Zool. 2005 Feb 16;2:3. doi: 10.1186/1742-9994-2-3 (PMC554092; doi:10.1186/1742-9994-2-3)
Supplement: Additional File 1 — Data matrix of characters – 79 taxa and 110 characters are included. N = non applicable. This implies that the character is not present and can therefore not be coded. ? = character state not known. Genus with an asterisk indicate that information on this genus is extracted from literature. Detailed information will be given elsewhere in Wägele & Klussmann-Kolb (in prep.). [file 1742-9994-2-3-S1.doc]

|  |  |  |  |  |  |  |  |  |  |  |  |  |  |  |  |  |  |  |  |  |  |  |  |  |  |  |  |  |  |  |
| --- | --- | --- | --- | --- | --- | --- | --- | --- | --- | --- | --- | --- | --- | --- | --- | --- | --- | --- | --- | --- | --- | --- | --- | --- | --- | --- | --- | --- | --- | --- |
| allzero | 0 | 0 | 0 | 0 | 0 | 0 | 0 | 0 | 0 | 0 | 0 | 0 | 0 | 0 | 0 | 0 | 0 | 0 | 0 | 0 | 0 | 0 | 0 | 0 | 0 | 0 | 0 | 0 | 0 | 0 |
| Valvata | 0 | 0 | 0 | 0 | 0 | 0 | 1 | 0 | 0 | 0 | 0 | 0 | 0 | 0 | 0 | 0 | 0 | 0 | 0 | 0 | 1 | 0 | 0 | 1 | 0 | 0 | 0 | 0 | 1 | 0 |
| Odostomia* | 0 | 0 | 0 | 0 | ? | 0 | 0 | 2 | 0 | 0 | 0 | 0 | 0 | 3 | 0 | 0 | 0 | 0 | 0 | 0 | 0 | 0 | 0 | ? | 0 | 0 | 0 | N | 0 | 0 |
| Pyramidella | 0 | 0 | 0 | 0 | 0 | 0 | 0 | 0 | 0 | 0 | 0 | 0 | 0 | 3 | 0 | 0 | 0 | 0 | 0 | 0 | 0 | 0 | 0 | ? | 0 | 0 | 0 | N | 0 | 0 |
| Heliacus* | 0 | 0 | 0 | 0 | ? | 0 | 1 | 2 | 0 | 0 | 0 | 0 | 0 | 0 | 0 | 0 | 0 | 0 | 0 | 0 | 0 | 0 | 0 | ? | 0 | 0 | 0 | N | 0 | 0 |
| Gegania* | 0 | 0 | 0 | 0 | ? | 0 | 1 | 0 | 0 | 0 | 0 | 0 | 0 | 0 | 0 | 0 | 0 | 0 | 0 | 0 | 0 | 0 | 0 | ? | 0 | 0 | 0 | 1 | 1 | 0 |
| Acteon | 0 | 0 | 0 | 0 | 0 | 0 | 0 | 0 | 0 | 1 | 0 | 1 | 0 | 3 | 0 | 0 | 0 | 0 | 0 | 0 | 0 | 1 | 0 | 1 | 0 | 0 | 0 | 0 | 1 | 0 |
| Pupa | 0 | 0 | 0 | 0 | 0 | 0 | 0 | 0 | 0 | 1 | 0 | 1 | 0 | 3 | ? | 0 | 0 | 0 | 0 | 0 | 0 | 1 | 0 | 1 | 0 | 0 | 0 | 0 | 1 | 0 |
| Bullina | 0 | 0 | 0 | 0 | 0 | 1 | 0 | 0 | 0 | 1 | 0 | 0 | 0 | 3 | 1 | 0 | 0 | 0 | 0 | 1 | 0 | 0 | 0 | 2 | 0 | 0 | 0 | 0 | 1 | 0 |
| Micromelo | 0 | 0 | 1 | 1 | 0 | 1 | 0 | 0 | 0 | 1 | 0 | 0 | 0 | 3 | 1 | 0 | 0 | 0 | 0 | 1 | 0 | 1 | 0 | 2 | 0 | 0 | 0 | 0 | 1 | 0 |
| Hydatina | 0 | 0 | 1 | 1 | 0 | 1 | 0 | 0 | 0 | 1 | 0 | 0 | 0 | 3 | 1 | 0 | 0 | 0 | 0 | 1 | 0 | 1 | 0 | 2 | 0 | 0 | 0 | 0 | 1 | 0 |
| Runcina | 1 | N | N | N | 0 | 0 | 0 | 0 | 0 | 0 | 0 | 0 | 0 | 3 | 1 | 0 | 0 | 0 | 0 | 2 | 0 | 0 | 1 | 0 | 0 | 0 | 0 | 0 | 1 | 0 |
| Metaruncina* | 2 | 2 | N | 1 | ? | 0 | 0 | 0 | 0 | 0 | 0 | 0 | 0 | 3 | 1 | 0 | 0 | 0 | 0 | 2 | 0 | 0 | 1 | 0 | 0 | 0 | 0 | 0 | 1 | 0 |
| Ringicula* | 0 | 0 | 0 | 1 | ? | ? | ? | ? | 0 | 1 | 0 | ? | 0 | 3 | 1 | 0 | 0 | 0 | 0 | 0 | 0 | 0 | 0 | 0 | 0 | 0 | 0 | 0 | 1 | 0 |
| Diaphana | 0 | 0 | 1 | 1 | ? | 1 | ? | 0 | 0 | 0 | 0 | ? | 0 | 3 | 1 | 0 | 0 | 0 | 0 | 1 | 0 | 0 | 0 | 0 | 0 | 0 | 0 | 0 | 1 | 0 |
| Newnesia | 0 | 2 | 0 | 1 | 0 | 1 | 0 | 2 | 0 | 0 | 0 | 0 | 0 | 3 | 1 | 0 | 0 | 0 | 0 | 1 | 0 | 0 | 0 | 0 | 0 | 0 | 0 | 0 | 1 | 0 |
| Colpodaspis | 0 | 2 | 1 | 1 | 0 | 1 | 0 | 2 | 0 | 0 | 0 | 0 | 0 | 3 | 1 | 0 | 0 | 0 | 0 | 1 | 0 | 0 | 0 | 0 | 0 | 0 | 0 | 0 | 1 | 0 |
| Bulla | 0 | 2 | 1 | 1 | 0 | 1 | 0 | 0 | 1 | 1 | 0 | 0 | 0 | 3 | 1 | 0 | 0 | 0 | 0 | 1 | 0 | 0 | 0 | 1 | 0 | 0 | 0 | 0 | 1 | 0 |
| Haminoea | 0 | 2 | 1 | 1 | 0 | 1 | 0 | 0 | 1 | 1 | 0 | 0 | 0 | 3 | 1 | 0 | 0 | 0 | 0 | 1 | 0 | 0 | 0 | 1 | 0 | 0 | 0 | 0 | 1 | 0 |
| Phanerophthalmus | 0 | 2 | 1 | 1 | 0 | ? | 0 | 0 | 1 | 1 | 0 | 0 | 0 | 3 | 1 | 0 | 0 | 0 | 0 | 1 | 0 | 0 | 0 | 0 | 0 | 0 | 0 | 0 | 1 | 0 |
| Smaragdinella | 0 | 2 | 1 | 1 | 0 | 1 | 0 | 0 | 1 | 1 | 0 | 2 | 0 | 3 | 1 | 0 | 0 | 0 | 0 | 1 | 0 | 0 | 0 | 0 | 0 | 0 | 0 | 0 | 1 | 0 |
| Siphopteron | 1 | N | N | N | 0 | 1 | 0 | 2 | 1 | 1 | 1 | 2 | 0 | 3 | 1 | 0 | 0 | 0 | 0 | 1 | 1 | 0 | 0 | 0 | 0 | 0 | 0 | 0 | 1 | 0 |
| Sagaminopteron | 2 | 2 | 1 | 1 | 0 | 1 | 0 | 2 | 1 | 1 | 1 | 2 | 0 | 3 | 1 | 0 | 0 | 0 | 0 | 1 | 1 | 0 | 0 | 0 | 0 | 0 | 0 | 0 | 1 | 0 |
| Gastropteron | 2 | 2 | 1 | 1 | 0 | 1 | 0 | 2 | 1 | 1 | 1 | 2 | 0 | 3 | 1 | 0 | 0 | 0 | 0 | 1 | ? | 0 | 0 | 0 | 0 | 0 | 0 | 0 | 1 | 0 |
| Philinopsis | 2 | 2 | 1 | 1 | 0 | 1 | 2 | 0 | 1 | 1 | 0 | 2 | 0 | 3 | 1 | 0 | 0 | 1 | 0 | 1 | 0 | 0 | 0 | 0 | 0 | 0 | 0 | 0 | 1 | 0 |
| Chelidonura | 2 | 2 | 1 | 1 | 0 | 1 | 2 | 0 | 1 | 1 | 0 | 2 | 0 | 3 | 1 | 0 | 0 | 1 | 0 | 1 | 0 | 0 | 0 | 0 | 0 | 0 | 0 | 0 | 1 | 0 |
| Philine | 2 | 2 | 1 | 1 | 0 | 1 | 0 | 0 | 1 | 1 | 0 | 2 | 0 | 3 | 1 | 0 | 0 | 0 | 0 | 1 | 0 | 0 | 0 | 1 | 0 | 0 | 0 | 0 | 1 | 0 |
| Cylichna | 0 | 2 | 1 | 1 | 0 | 0 | 0 | 0 | 0 | 1 | 0 | 0 | 0 | 3 | 1 | 0 | 0 | 0 | 0 | 1 | 0 | 0 | 0 | 1 | 0 | 0 | 0 | 0 | 1 | 0 |
| Retusa | 0 | 0 | 1 | 1 | ? | 0 | ? | ? | 0 | 1 | 0 | 0 | 0 | 3 | 1 | 0 | 0 | 0 | 0 | 1 | 0 | 0 | 0 | 1 | 0 | 0 | 0 | N | 1 | 0 |
| Scaphander | 0 | 0 | 1 | 1 | 0 | 1 | 0 | 0 | 1 | 1 | 0 | 2 | 0 | 3 | 1 | 0 | 0 | 0 | 0 | 1 | 0 | 1 | 0 | 1 | 0 | 0 | 0 | 0 | 1 | 0 |
| Acteocina | 0 | 0 | 0 | 1 | 0 | 0 | ? | ? | 0 | 1 | 0 | 0 | 0 | 3 | ? | 0 | 0 | 0 | 0 | 1 | 0 | 0 | 0 | ? | 0 | 0 | 0 | 0 | 1 | 0 |
| Akera | 0 | 0 | 1 | 1 | 0 | 1 | 0 | 0 | 1 | 0 | 0 | 0 | 0 | 3 | 1 | 0 | 0 | 0 | 0 | 1 | 0 | 1 | 0 | 0 | 0 | 0 | 0 | 0 | 1 | 0 |
| Aplysia | 2 | 2 | 1 | 1 | 0 | 1 | 0 | 2 | 1 | 0 | 0 | 0 | 1 | 3 | 1 | 1 | 0 | 0 | 0 | 1 | 1 | 0 | 0 | 0 | 0 | 0 | 0 | 0 | 1 | 0 |
| Dolabella | 2 | 2 | 1 | 1 | 0 | 1 | 0 | 0 | 1 | 0 | 0 | 0 | 1 | 3 | 1 | 1 | 0 | 0 | 0 | 1 | 1 | 0 | 0 | 0 | 0 | 0 | 0 | 0 | 1 | 0 |
| Cylindrobulla* | 0 | 0 | 1 | 1 | ? | 0 | ? | ? | 0 | 1 | 0 | 1 | 0 | 3 | 1 | 0 | 0 | 0 | 0 | 1 | 0 | 0 | 0 | 1 | 0 | 0 | 0 | 1 | 2 | 0 |
| Ascobulla* | 0 | 0 | 1 | 1 | ? | 0 | ? | ? | 0 | 1 | 0 | 1 | 0 | 3 | 1 | 0 | 0 | 0 | 0 | 1 | 0 | 0 | 0 | ? | 0 | 0 | 0 | 1 | 2 | 0 |
| Elysia | 1 | N | N | N | 0 | 0 | 0 | 0 | 1 | 0 | 0 | 0 | 1 | 3 | 1 | 0 | 0 | 0 | 2 | N | 1 | N | 1 | 0 | 0 | 0 | 0 | N | 0 | 0 |
| Oxynoe | 0 | 2 | 1 | 1 | 0 | 0 | 0 | 0 | 1 | 0 | 0 | 0 | 1 | 3 | 1 | 0 | 0 | 0 | 0 | 1 | ? | 0 | 0 | 2 | 0 | 0 | 0 | 1 | 2 | 0 |
| Lobiger | 0 | 2 | 1 | 1 | 0 | 0 | ? | ? | 1 | 0 | 0 | 0 | 1 | 3 | 1 | 1 | 0 | 0 | 0 | 1 | 1 | 0 | 0 | 2 | 0 | 0 | 0 | 1 | 2 | 0 |
| Tylodina | 0 | 1 | 0 | 1 | 0 | 0 | 0 | 0 | 0 | 0 | 0 | 0 | 1 | 3 | 1 | 1 | 0 | 0 | 2 | N | 1 | N | 0 | 2 | 0 | 0 | 0 | 0 | 1 | 0 |
| Umbraculum | 0 | 1 | 0 | 1 | 0 | 0 | 0 | 0 | 0 | 0 | 0 | 0 | 1 | 3 | 1 | 0 | 0 | 0 | 2 | N | 1 | N | 0 | 2 | 0 | 0 | 0 | 0 | 1 | 0 |
| Hedylopsis* | 1 | N | N | N | ? | 0 | 0 | 0 | 0 | 0 | 0 | 0 | 2 | 3 | 0 | 2 | 0 | 0 | 2 | 0 | 1 | 0 | 1 | 1 | 0 | 0 | 0 | N | 0 | 0 |
| Microhedyle* | 1 | N | N | N | ? | 0 | 0 | 0 | 0 | 0 | 0 | 0 | 2 | 3 | 0 | 2 | 0 | 0 | 2 | 0 | 1 | 0 | 1 | 1 | 0 | 0 | 0 | N | 0 | 0 |
| Unela* | 1 | N | N | N | ? | 0 | 0 | 0 | 0 | 0 | 0 | 0 | 2 | 3 | 0 | 2 | 0 | 0 | 2 | 0 | 1 | 0 | 1 | 1 | 0 | 0 | 0 | N | 0 | 0 |
| Philinoglossa* | 1 | N | N | 1 | ? | 0 | 0 | 0 | 0 | 0 | 0 | 0 | 0 | 3 | 1 | 0 | 0 | 0 | 2 | N | 1 | N | 1 | 0 | 0 | 0 | 0 | N | 0 | 0 |
| Pluscula* | 2 | 1 | ? | 1 | ? | 0 | 0 | 0 | 0 | 0 | 0 | 0 | 0 | 3 | 1 | 0 | 0 | 0 | 2 | N | 1 | N | 1 | 0 | 0 | 0 | 0 | N | 0 | 0 |
| Philinoglossa_nov | 1 | N | N | 1 | 0 | 0 | 0 | 0 | 0 | 0 | 0 | 0 | 0 | 3 | 1 | 0 | 0 | 0 | 2 | N | 1 | N | 1 | 0 | 0 | 0 | 0 | N | 0 | 0 |
| Tomthompsonia | 2 | 0 | 0 | 1 | 0 | 0 | 0 | 1 | 0 | 0 | 0 | 0 | 1 | 3 | 1 | 1 | 0 | 0 | 2 | N | 1 | N | 0 | 0 | 0 | 0 | 0 | 0 | 1 | 0 |
| Berthella | 2 | 2 | 0 | 1 | 0 | 0 | 0 | 0 | 0 | 0 | 0 | 0 | 1 | 3 | 1 | 1 | 0 | 0 | 2 | N | 1 | N | 0 | 0 | 0 | 0 | 0 | 0 | 1 | 0 |
| Bathyberthella | 2 | 2 | 0 | 1 | 0 | 0 | 0 | 1 | 0 | 0 | 0 | 0 | 1 | 3 | 1 | 1 | 0 | 0 | 2 | N | 1 | N | 0 | 0 | 0 | 0 | 0 | 0 | 1 | 0 |
| Bathydoris | 1 | N | N | N | 0 | 0 | 0 | 0 | 0 | 0 | 0 | 0 | 3 | 3 | 1 | 2 | 0 | 0 | 2 | N | 1 | N | 0 | 0 | 0 | 0 | 0 | 0 | 1 | 1 |
| Goniodoris | 1 | N | N | N | 0 | 0 | 0 | 0 | 0 | 0 | 0 | 0 | 3 | 3 | 1 | 0 | 0 | 0 | 2 | N | 1 | N | 0 | 0 | 0 | 0 | 0 | 0 | 1 | 1 |
| Jorunna | 1 | N | N | N | 0 | 0 | 0 | 0 | 0 | 0 | 0 | 0 | 3 | 3 | 1 | 0 | 0 | 0 | 2 | N | 1 | N | 0 | 0 | 0 | 0 | 0 | 0 | 1 | 1 |
| Austrodoris | 1 | N | N | N | 0 | 0 | 0 | 0 | 0 | 0 | 0 | 0 | 3 | 3 | 1 | 0 | 0 | 0 | 2 | N | 1 | N | 0 | 0 | 0 | 0 | 0 | 0 | 1 | 1 |
| Polycera | 1 | N | N | N | 0 | 0 | 0 | 0 | 0 | 0 | 0 | 0 | 3 | 3 | 1 | 0 | 0 | 0 | 2 | N | 1 | N | 0 | 2 | 1 | 0 | 0 | 0 | 1 | 1 |
| Limacia | 1 | N | N | N | 0 | 0 | 0 | 0 | 0 | 0 | 0 | 0 | 3 | 3 | 1 | 0 | 0 | 0 | 2 | N | 1 | N | 0 | 2 | 0 | 0 | 0 | 0 | 1 | 1 |
| Rhodope* | 1 | N | N | N | 0 | 0 | 0 | 1 | 0 | 0 | 0 | 0 | 0 | 3 | ? | 0 | 0 | 0 | 2 | N | 1 | N | 1 | 0 | 1 | 0 | 0 | N | 0 | 0 |
| Helminthope* | 1 | N | N | N | 0 | 0 | 0 | 0 | 0 | 0 | 0 | 0 | 0 | 3 | ? | 0 | 0 | 0 | 2 | N | 1 | N | 1 | 0 | 1 | 0 | 0 | N | 0 | 0 |
| Doridoxa | 1 | N | N | N | 0 | 0 | 0 | ? | 0 | 0 | 0 | 0 | 3 | 3 | 1 | 0 | 0 | 0 | 2 | N | 1 | N | 0 | 0 | 0 | 0 | 0 | N | 0 | 0 |
| Armina | 1 | N | N | N | 0 | 0 | 0 | 1 | 0 | 0 | 0 | 0 | 3 | 3 | 1 | 0 | 0 | 0 | 2 | N | 1 | N | 0 | 0 | 0 | 0 | 0 | N | 0 | 0 |
| Dermatobranchus | 1 | N | N | N | 0 | 0 | 0 | 1 | 0 | 0 | 0 | 0 | 3 | 3 | 1 | 0 | 0 | 0 | 2 | N | 1 | N | 0 | 0 | 0 | 0 | 0 | N | 0 | 0 |
| Dendronotus | 1 | N | N | N | 0 | 0 | 0 | 0 | 0 | 0 | 0 | 0 | 3 | 3 | 1 | 0 | 0 | 0 | 2 | N | 1 | N | 1 | 0 | 1 | 0 | 1 | N | 0 | 0 |
| Tritonia | 1 | N | N | N | 0 | 0 | 0 | 0 | 0 | 0 | 0 | 0 | 3 | 3 | 1 | 0 | 0 | 0 | 2 | N | 1 | N | 1 | 0 | 0 | 0 | 1 | N | 0 | 0 |
| Pseudotritonia | 1 | N | N | N | 0 | 0 | 0 | 0 | 0 | 0 | 0 | 0 | 3 | 3 | ? | 0 | 0 | 0 | 2 | N | 1 | N | 1 | 0 | 0 | 0 | 0 | N | 0 | 0 |
| Charcotia | 1 | N | N | N | 0 | 0 | 0 | 0 | 0 | 0 | 0 | 0 | 3 | 3 | ? | 0 | 0 | 0 | 2 | N | 1 | N | 1 | 0 | 0 | 0 | 0 | N | 0 | 0 |
| Notaeolidia | 1 | N | N | N | 0 | 0 | 0 | 0 | 0 | 0 | 0 | 0 | 3 | 3 | 1 | 2 | 0 | 0 | 2 | N | 1 | N | 1 | 0 | 0 | 1 | 0 | N | 0 | 0 |
| Flabellina | 1 | N | N | N | 0 | 0 | 0 | 0 | 0 | 0 | 0 | 0 | 3 | 3 | 1 | 2 | 1 | 0 | 2 | N | 1 | N | 1 | 0 | 1 | 1 | 0 | N | 0 | 0 |
| Aeolidia | 1 | N | N | N | 0 | 0 | 0 | 0 | 0 | 0 | 0 | 0 | 3 | 3 | 1 | 2 | 1 | 0 | 2 | N | 1 | N | 1 | 0 | 1 | 1 | 0 | N | 0 | 0 |
| Onchidium | 1 | N | N | N | 1 | 0 | 1 | 0 | 0 | 0 | 0 | 0 | 0 | 1 | 0 | 0 | 0 | 0 | 1 | 2 | 1 | 0 | 0 | 0 | 0 | 0 | 0 | N | 0 | 0 |
| Onchidella | 1 | N | N | N | 1 | 0 | 1 | 0 | 0 | 0 | 0 | 0 | 0 | 1 | 0 | 0 | 0 | 0 | 1 | 2 | 1 | 0 | 0 | 0 | 0 | 0 | 0 | N | 0 | 0 |
| Siphonaria | 0 | 1 | 0 | 1 | 1 | 0 | 0 | ? | 0 | 0 | 0 | 0 | 0 | 3 | 0 | 0 | 0 | 0 | 1 | 1 | 0 | 0 | 0 | 1 | 0 | 0 | 0 | 0 | 0 | 0 |
| Ellobiidae | 0 | 0 | 0 | 0 | 0 | 0 | 1 | 0 | 0 | 0 | 0 | 0 | 0 | ? | 0 | 0 | 0 | 0 | 1 | 1 | 0 | 0 | 0 | 1 | 0 | 0 | 0 | N | 0 | 0 |
| Physa | 0 | 0 | 0 | 1 | 1 | 0 | 0 | 0 | 0 | 0 | 0 | 0 | 0 | 3 | 0 | 0 | 0 | 0 | 1 | 1 | 1 | 0 | 0 | 1 | 0 | 0 | 0 | N | 0 | 0 |
| Ancylus | 0 | 1 | 0 | 1 | 0 | 0 | 0 | 0 | 0 | 0 | 0 | 0 | 0 | 3 | 0 | 0 | 0 | 0 | 2 | N | 1 | N | 0 | 1 | 0 | 0 | 0 | N | 0 | 0 |
| Amphibola | 0 | 0 | 0 | 0 | ? | 0 | ? | ? | 0 | 0 | 0 | 0 | 0 | ? | 0 | 0 | 0 | 0 | 1 | 1 | 0 | 0 | 0 | 1 | 0 | 0 | 0 | N | 0 | 0 |
| Cepaea | 0 | 0 | 0 | 1 | 0 | 0 | 1 | 0 | 0 | 0 | 0 | 0 | 0 | 2 | 0 | 2 | 0 | 0 | 1 | 1 | 1 | 0 | 0 | 0 | 0 | 0 | 0 | N | 0 | 0 |
| Limacina | 0 | 0 | 0 | 0 | 0 | 1 | ? | 2 | 1 | 0 | 0 | 0 | 0 | 3 | 0 | 0 | 0 | 0 | 0 | 0 | ? | 0 | 0 | 1 | 0 | 0 | 0 | N | 0 | 0 |
| Cavolinia | 0 | 3 | 0 | 1 | 0 | 1 | 0 | 0 | 1 | 0 | 0 | 0 | 0 | 4 | 0 | 0 | 0 | 0 | 0 | 0 | ? | 0 | 0 | 1 | 0 | 0 | 0 | 0 | 2 | 0 |
| Spongiobranchaea | 1 | N | N | N | 0 | 0 | 0 | 0 | 1 | 0 | 0 | 0 | 0 | 4 | 0 | 2 | 0 | 0 | 2 | N | 1 | N | 1 | 1 | 0 | 0 | 0 | N | 0 | 0 |
| Clione | 1 | N | N | N | 0 | 0 | 0 | 1 | 1 | 0 | 0 | 0 | 0 | 4 | 0 | 2 | 0 | 0 | 2 | N | 1 | N | 1 | 1 | 0 | 0 | 0 | N | 0 | 0 |

|  |  |  |  |  |  |  |  |  |  |  |  |  |  |  |  |  |  |  |  |  |  |  |  |  |  |  |  |  |  |  |
| --- | --- | --- | --- | --- | --- | --- | --- | --- | --- | --- | --- | --- | --- | --- | --- | --- | --- | --- | --- | --- | --- | --- | --- | --- | --- | --- | --- | --- | --- | --- |
| allzero | 0 | 0 | 0 | 0 | 0 | 0 | 0 | 0 | 0 | 0 | 0 | 0 | 0 | 0 | 0 | 0 | 0 | 0 | 0 | 0 | 0 | 0 | 0 | 0 | 0 | 0 | 0 | 0 | 0 | 0 |
| Valvata | 0 | 0 | 0 | 0 | 0 | 0 | 0 | 0 | 0 | 0 | 0 | 0 | 0 | 0 | 0 | 0 | 0 | 0 | 0 | 0 | 0 | 5 | 0 | 0 | 0 | 0 | 0 | 0 | 0 | 0 |
| Odostomia* | 0 | 0 | 0 | 0 | 2 | 0 | 0 | 0 | 0 | 0 | 1 | 0 | 0 | 2 | 0 | 0 | 1 | N | N | N | N | N | 0 | 0 | ? | 0 | 0 | 0 | 0 | 0 |
| Pyramidlla | 0 | 1 | 0 | 0 | 2 | 0 | 0 | 0 | 0 | 0 | 1 | 0 | 0 | 2 | 0 | 0 | 1 | N | N | N | N | N | 0 | 0 | 0 | 0 | 0 | 0 | 0 | 0 |
| Heliacus* | 0 | 1 | 0 | 0 | 1 | 0 | 0 | 0 | 0 | 0 | 0 | 0 | 0 | 0 | 0 | 0 | 0 | 0 | 0 | 0 | 0 | 0 | 0 | 0 | 2 | 0 | 0 | 0 | 0 | 0 |
| Gegania* | 0 | 1 | 0 | 0 | 1 | 0 | 0 | 0 | 0 | 0 | 0 | 0 | 1 | N | 0 | 0 | 0 | 0 | 0 | 0 | 0 | ? | 0 | 0 | 1 | 0 | 0 | 0 | 0 | 0 |
| Acteon | 0 | 0 | 0 | 0 | 0 | 1 | 1 | 0 | 0 | 0 | 0 | 0 | 1 | N | 0 | 0 | 0 | 1 | N | N | 0 | 5 | 0 | 0 | 2 | 0 | 0 | 0 | 0 | 0 |
| Pupa | 0 | 0 | ? | 0 | 0 | 0 | 0 | 0 | 0 | 0 | 0 | 0 | 0 | 0 | 0 | 0 | 0 | 0 | ? | 0 | 0 | 5 | 0 | 0 | 2 | 0 | 0 | 0 | 0 | 0 |
| Bullina | 0 | 0 | ? | 0 | 0 | 0 | 0 | 0 | 1 | 0 | 0 | 0 | 0 | 0 | 0 | 0 | 0 | 0 | 3 | 0 | 0 | 5 | 0 | 0 | 1 | 0 | 0 | 0 | 0 | 0 |
| Micromelo | 0 | 0 | 0 | 0 | 0 | 0 | 0 | 0 | 1 | 0 | 0 | 0 | 0 | 0 | 0 | 0 | 0 | 0 | 3 | 0 | 0 | 5 | 0 | 0 | 1 | 0 | 0 | 0 | 0 | 0 |
| Hydatina | 0 | 0 | ? | 0 | 0 | 0 | 0 | 0 | 1 | 0 | 0 | 0 | 0 | 0 | 0 | 0 | 0 | 0 | 5 | 0 | 0 | 5 | 0 | 0 | 1 | 0 | 0 | 0 | 0 | 0 |
| Runcina | 0 | 0 | 1 | 2 | 0 | 0 | 0 | 0 | 0 | 0 | 0 | 0 | 0 | 0 | 0 | 0 | 0 | 0 | 1 | 0 | 0 | 2 | 0 | 0 | 3 | 1 | 2 | 0 | 0 | 0 |
| Metaruncina* | 0 | 0 | ? | 2 | 0 | ? | 1 | 0 | 0 | 0 | 0 | 0 | 0 | 0 | 0 | 0 | 0 | ? | ? | 0 | ? | ? | 0 | 0 | 3 | 1 | 2 | 0 | 0 | 0 |
| Ringicula* | 0 | 0 | 0 | 0 | 0 | 1 | 1 | 0 | 0 | 0 | 0 | 0 | 1 | N | N | 0 | 0 | 1 | N | N | 0 | 2 | 0 | 0 | 0 | 0 | 0 | 0 | 0 | ? |
| Diaphana | 0 | 0 | ? | 0 | 0 | ? | 0 | 0 | 0 | 0 | 0 | 0 | 1 | N | N | 0 | 0 | 0 | 1 | 0 | 0 | 0 | 0 | ? | ? | 0 | 0 | 0 | 0 | ? |
| Newnesia | 0 | 0 | 0 | 0 | 0 | 2 | 0 | 0 | 0 | 0 | 1 | 0 | 1 | N | N | 0 | 0 | 0 | 0 | 0 | 1 | N | 0 | 0 | 2 | 0 | 0 | 0 | 2 | 0 |
| Colpodaspis | 0 | 0 | ? | 0 | 0 | 0 | 0 | 0 | 0 | 0 | 0 | 0 | 1 | N | N | 0 | 0 | 1 | N | N | 0 | 0 | 0 | 0 | 0 | 0 | 0 | 0 | 0 | 0 |
| Bulla | 0 | 0 | 0 | 2 | 0 | 1 | 0 | 0 | 0 | 0 | 0 | 0 | 0 | 0 | 0 | 0 | 0 | 0 | 5 | 0 | 0 | 5 | 0 | 0 | 3 | 1 | 1 | 0 | 2 | 0 |
| Haminoea | 0 | 0 | 0 | 2 | 0 | 0 | 0 | 0 | 0 | 0 | 0 | 0 | 0 | 0 | 0 | 0 | 0 | 0 | 0 | 0 | 0 | 0 | 0 | 0 | 3 | 1 | 1 | 3 | 2 | 0 |
| Phanerophthalmus | 0 | 0 | 0 | 2 | 0 | 0 | 0 | 0 | 0 | 0 | 0 | 0 | 0 | 0 | 0 | 0 | 0 | 0 | 0 | 0 | 0 | 0 | 0 | 0 | 3 | 1 | 1 | 0 | 2 | 0 |
| Smaragdinella | 0 | 0 | ? | 2 | 0 | 0 | 0 | 0 | 0 | 0 | 0 | 0 | 0 | 0 | 0 | 0 | 0 | 0 | 0 | 0 | 0 | 0 | 0 | 0 | 3 | 1 | 1 | 3 | 2 | 0 |
| Siphopteron | 0 | 0 | 0 | 0 | 0 | 0 | 0 | 0 | 0 | 0 | 0 | 0 | 0 | 0 | 0 | 0 | 0 | 1 | N | N | 0 | 1 | 0 | 0 | 0 | 0 | 0 | 0 | 0 | 0 |
| Sagaminopteron | 0 | 0 | 0 | 0 | 0 | 0 | 0 | 0 | 0 | 0 | 0 | 0 | 1 | N | N | 0 | 0 | 1 | N | N | 0 | 1 | 0 | 0 | 0 | 0 | 0 | 0 | 0 | 0 |
| Gastropteron | 0 | 0 | 0 | 0 | 0 | 0 | 0 | 0 | 0 | 0 | 0 | 0 | 0 | 0 | 0 | 0 | 0 | 1 | N | N | 0 | 1 | 0 | 0 | 0 | 0 | 0 | 0 | 0 | 0 |
| Philinopsis | 0 | 0 | 0 | 2 | 0 | 0 | 0 | 0 | 0 | 0 | 0 | 0 | 1 | N | N | 0 | 1 | N | N | N | N | N | 0 | 0 | 0 | 0 | 0 | 0 | 0 | 0 |
| Chelidonura | 0 | 0 | 0 | 2 | 0 | 0 | 0 | 0 | 0 | 0 | 0 | 0 | 1 | N | N | 0 | 1 | N | N | N | N | N | 0 | 0 | 0 | 0 | 0 | 0 | 0 | 0 |
| Philine | 0 | 0 | 0 | 2 | 0 | 0 | 0 | 0 | 0 | 0 | 0 | 0 | 1 | N | N | 0 | 0 | 0 | N | N | 0 | 2 | 0 | 0 | 3 | 1 | 1 | 2 | 2 | 0 |
| Cylichna | 0 | 0 | 0 | 0 | 0 | 0 | 0 | 0 | 0 | 0 | 0 | 0 | 0 | 0 | 0 | 0 | 0 | 0 | 1 | 0 | 0 | 1 | 0 | 0 | 3 | 1 | 1 | 2 | 0 | 0 |
| Retusa | 0 | 0 | 0 | 0 | 0 | 0 | 0 | 0 | 0 | 0 | 0 | 0 | 1 | N | N | 0 | 1 | ? | N | N | N | N | 0 | 0 | ? | 1 | 1 | 1 | ? | 0 |
| Scaphander | 0 | 0 | 0 | 2 | 0 | 0 | 0 | 0 | 0 | 0 | 0 | 0 | 1 | N | N | 0 | 0 | 0 | 0 | 0 | 0 | 2 | 0 | 0 | 3 | 1 | 1 | 2 | 0 | 0 |
| Acteocina | 0 | 0 | ? | 0 | 0 | 0 | ? | 0 | 0 | 0 | 0 | 0 | 0 | 0 | 0 | 0 | 0 | 0 | 1 | 0 | 0 | 2 | 0 | ? | ? | 1 | 1 | 2 | 2 | 0 |
| Akera | 0 | 0 | 0 | 0 | 0 | 0 | 0 | 0 | 0 | 0 | 0 | 0 | 0 | 0 | 0 | 0 | 0 | 0 | 0 | 0 | 0 | 0 | 1 | 0 | 3 | 1 | 0 | 2 | 2 | 0 |
| Aplysia | 0 | 0 | 0 | 2 | 0 | 0 | 0 | 0 | 0 | 1 | 0 | 0 | 0 | 0 | 0 | 0 | 0 | 0 | 0 | 0 | 0 | 0 | 1 | 0 | 3 | 1 | 0 | 2 | 2 | 0 |
| Dolabella | 0 | 0 | 0 | 2 | 0 | 0 | 0 | 0 | 0 | 1 | 0 | 0 | 0 | 0 | 0 | 0 | 0 | 0 | 0 | 0 | 0 | 0 | 1 | 0 | 3 | 1 | 0 | 2 | 2 | 0 |
| Cylindrobulla* | 0 | 0 | 0 | 0 | 0 | 2 | 0 | 0 | 0 | 0 | 0 | 1 | 1 | N | N | 1 | 0 | 0 | 0 | 0 | 1 | N | 0 | 0 | 0 | 0 | 0 | 0 | 0 | ? |
| Ascobulla* | 0 | 0 | ? | ? | 0 | 2 | 0 | 0 | 0 | 0 | 0 | 1 | 1 | N | N | 1 | 0 | 0 | 2 | 1 | 1 | N | 0 | 0 | ? | 0 | 0 | 0 | 0 | ? |
| Elysia | 0 | 0 | 1 | 0 | 0 | 0 | 0 | 0 | 0 | 0 | 0 | 0 | 1 | N | N | 1 | 0 | 0 | 2 | 1 | 1 | N | 0 | 1 | 0 | 0 | 0 | 0 | 0 | 0 |
| Oxynoe | 0 | 0 | 0 | 2 | 0 | 0 | 0 | 0 | 0 | 0 | 0 | 1 | 1 | N | N | 1 | 0 | 0 | 2 | 1 | 1 | N | 0 | 0 | 0 | 0 | 0 | 0 | 0 | 0 |
| Lobiger | 0 | 0 | 0 | 2 | 0 | ? | 0 | 0 | 0 | 0 | 0 | 1 | 1 | N | N | 1 | 0 | 0 | 2 | 1 | 1 | N | 0 | 0 | ? | 0 | 0 | 0 | 0 | 0 |
| Tylodina | 0 | 0 | 0 | 0 | 0 | 2 | 0 | 0 | 0 | 0 | 0 | 0 | 1 | N | N | 0 | 0 | 0 | 5 | 0 | 0 | 0 | 0 | 0 | 3 | 0 | 0 | 0 | 1 | 0 |
| Umbraculum | 0 | 0 | ? | 0 | 0 | 2 | 0 | 0 | 0 | 0 | 0 | 0 | 1 | N | N | 0 | 0 | 1 | N | N | 0 | 0 | 0 | 0 | 3 | 0 | 0 | 0 | 1 | 0 |
| Hedylopsis* | 0 | 0 | ? | 0 | 0 | 0 | 0 | 0 | 0 | 0 | 0 | 0 | 1 | N | N | 0 | 0 | 0 | 0 | 0 | 0 | 4 | 0 | 0 | 0 | 0 | 0 | 0 | 0 | 0 |
| Microhedyle* | 0 | 0 | 1 | 0 | 0 | 0 | 0 | 0 | 0 | 0 | 0 | 0 | 1 | N | N | 0 | 0 | 0 | 0 | 0 | 0 | 4 | 0 | 0 | 0 | 0 | 0 | 0 | 0 | 0 |
| Unela* | 0 | 0 | ? | 0 | 0 | 0 | 2 | 0 | 0 | 0 | 0 | 0 | 1 | N | N | 0 | 0 | 0 | 0 | 0 | 0 | 4 | 0 | 0 | 0 | 0 | 0 | 0 | 0 | 0 |
| Philinoglossa* | 0 | 0 | 1 | 2 | 0 | 0 | 1 | 0 | 0 | 0 | 0 | 0 | 1 | N | N | 0 | 0 | 1 | N | N | 0 | 0 | 0 | 0 | 0 | 0 | 0 | 0 | 0 | 0 |
| Pluscula* | 0 | 0 | ? | 2 | 0 | 0 | 1 | 0 | 0 | 0 | 0 | 0 | 1 | N | N | 0 | 0 | 1 | N | N | 0 | ? | 0 | 0 | 0 | 0 | 0 | 0 | 0 | 0 |
| Philinoglossa_nov | 0 | 0 | 1 | 2 | 0 | 0 | 0 | 0 | 0 | 0 | 0 | 0 | 1 | N | N | 0 | 0 | 1 | N | N | 0 | 0 | 0 | 0 | 3 | 1 | 1 | 2 | 0 | 0 |
| Tomthompsonia | 0 | 0 | 1 | 0 | 0 | 0 | 0 | 0 | 0 | 0 | 0 | 0 | 0 | 0 | 0 | 0 | 0 | 1 | N | N | 0 | 0 | 0 | 1 | 0 | 0 | 0 | 0 | 0 | 0 |
| Berthella | 0 | 0 | 1 | 0 | 0 | 0 | 0 | 1 | 0 | 0 | 0 | 0 | 0 | 0 | 0 | 0 | 0 | 1 | N | N | 0 | 0 | 0 | 1 | 0 | 0 | 0 | 0 | 0 | 0 |
| Bathyberthella | 0 | 0 | 1 | 0 | 0 | 0 | 0 | 1 | 0 | 0 | 0 | 0 | 0 | 0 | 0 | 0 | 0 | 1 | N | N | 0 | 0 | 0 | 1 | 2 | 0 | 0 | 0 | 0 | 0 |
| Bathydoris | 0 | 0 | 1 | 1 | 0 | 1 | 0 | 0 | 0 | 0 | 0 | 0 | 0 | 1 | 1 | 0 | 0 | 0 | 0 | 0 | 0 | 0 | 0 | 1 | 2 | 0 | 0 | 0 | 1 | 0 |
| Goniodoris | 0 | 0 | 1 | 1 | 0 | 0 | 0 | 0 | 0 | 0 | 1 | 0 | 0 | 0 | 0 | 0 | 0 | 1 | N | N | 0 | 3 | 0 | 0 | 0 | 0 | 0 | 0 | 0 | 0 |
| Jorunna | 1 | 0 | 1 | 1 | 0 | 0 | 0 | 0 | 0 | 0 | 0 | 0 | 1 | N | N | 0 | 0 | 0 | N | N | 0 | 0 | 0 | 0 | 0 | 0 | 0 | 0 | 0 | 0 |
| Austrodoris | 1 | 0 | 1 | 1 | 0 | 0 | 0 | 0 | 0 | 0 | 0 | 0 | 1 | N | N | 0 | 0 | 1 | N | N | 0 | 0 | 0 | 0 | 0 | 0 | 0 | 0 | 0 | 0 |
| Polycera | 0 | 0 | 1 | 1 | 0 | 0 | 0 | 0 | 0 | 0 | 0 | 0 | 0 | 1 | 1 | 0 | 0 | 1 | N | N | 0 | 3 | 0 | 0 | 0 | 0 | 0 | 0 | 0 | 0 |
| Limacia | 0 | 0 | 1 | 1 | 0 | 0 | 0 | 0 | 0 | 0 | 0 | 0 | 1 | N | N | 0 | 0 | 1 | N | N | 0 | 3 | 0 | 0 | 0 | 0 | 0 | 0 | 0 | 0 |
| Rhodope | 0 | 0 | ? | 0 | 0 | 0 | 1 | 0 | 0 | 0 | 0 | 0 | 1 | N | N | 0 | 1 | N | N | N | N | N | 0 | 0 | 0 | 0 | 0 | 0 | 0 | 0 |
| Helminthope | 0 | 0 | ? | 0 | 0 | ? | 0 | 0 | 0 | 0 | 0 | 0 | 1 | N | N | 0 | 1 | N | N | N | N | N | 0 | 0 | 0 | 0 | 0 | 0 | 0 | 0 |
| Doridoxa | 0 | 0 | 1 | 0 | 0 | 0 | 0 | 0 | 0 | 0 | 0 | 0 | 0 | 1 | 0 | 0 | 0 | 0 | 0 | 0 | 0 | 0 | 0 | 0 | ? | 0 | 0 | 0 | 0 | 0 |
| Armina | 0 | 0 | 1 | 0 | 0 | 0 | 2 | 0 | 0 | 0 | 0 | 0 | 0 | 1 | 0 | 0 | 0 | 0 | 0 | 0 | 0 | 0 | 0 | 0 | 1 | 0 | 0 | 0 | 0 | 0 |
| Dermatobranchus | 0 | 0 | 1 | 0 | 0 | 0 | 0 | 0 | 0 | 0 | 0 | 0 | 0 | 1 | 0 | 0 | 0 | 0 | 0 | 0 | 0 | 0 | 0 | 0 | 1 | 0 | 0 | 0 | 0 | 0 |
| Dendronotus | 0 | 0 | 1 | 0 | 0 | 0 | 0 | 0 | 0 | 0 | 0 | 0 | 0 | 1 | 0 | 0 | 0 | 0 | 0 | 0 | 0 | 0 | 0 | 0 | 1 | 0 | 0 | 0 | 0 | 0 |
| Tritonia | 0 | 0 | 1 | 0 | 0 | 0 | 0 | 0 | 0 | 0 | 0 | 0 | 0 | 1 | 0 | 0 | 0 | 0 | 0 | 0 | 0 | 0 | 0 | 0 | 1 | 0 | 0 | 0 | 0 | 0 |
| Pseudotritonia | 0 | 0 | 1 | 0 | 0 | 0 | 0 | 0 | 0 | 0 | 0 | 0 | 0 | 1 | 0 | 0 | 0 | 0 | 0 | 0 | 0 | 4 | 0 | 0 | 1 | 0 | 0 | 0 | 0 | 0 |
| Charcotia | 0 | 0 | 1 | 0 | 0 | 0 | 0 | 0 | 0 | 0 | 0 | 0 | 0 | 1 | 0 | 0 | 0 | 0 | 0 | 0 | 0 | 4 | 0 | 0 | 1 | 0 | 0 | 0 | 0 | 0 |
| Notaeolidia | 0 | 0 | 1 | 0 | 0 | 0 | 0 | 0 | 0 | 0 | 0 | 0 | 0 | 1 | 1 | 0 | 0 | 0 | 0 | 0 | 0 | 0 | 0 | 0 | 1 | 0 | 0 | 0 | 0 | 0 |
| Flabellina | 0 | 0 | 1 | 0 | 0 | 0 | 1 | 0 | 0 | 0 | 0 | 0 | 0 | 1 | 0 | 0 | 0 | 0 | 0 | 0 | 0 | 2 | 0 | 0 | 1 | 0 | 0 | 0 | 0 | 0 |
| Aeolidia | 0 | 0 | 1 | 0 | 0 | 0 | 0 | 0 | 0 | 0 | 0 | 0 | 0 | 1 | 1 | 0 | 0 | 0 | 4 | 0 | 1 | N | 0 | 0 | 1 | 0 | 0 | 0 | 0 | 0 |
| Onchidium | 0 | 0 | 1 | 3 | 0 | 0 | 0 | 0 | 0 | 0 | 0 | 0 | 1 | N | N | 0 | 0 | 0 | 5 | 0 | 0 | 0 | 0 | 0 | 1 | 0 | 0 | 0 | 0 | 1 |
| Onchidella | 0 | 0 | 1 | 3 | 0 | 0 | 0 | 0 | 0 | 0 | 0 | 0 | 1 | N | N | 0 | 0 | 0 | 5 | 0 | 0 | 0 | 0 | 0 | 1 | 0 | 0 | 0 | 0 | 1 |
| Siphonaria | 0 | 1 | 0 | 0 | 0 | 0 | 0 | 0 | 0 | 0 | 0 | 0 | 2 | 0 | ? | 0 | 0 | 0 | 0 | 0 | 0 | 0 | 0 | 0 | 0 | 0 | 0 | 0 | 0 | 0 |
| Ellobiidae | 0 | 0 | 1 | 0 | 0 | 0 | 0 | 0 | 0 | 0 | 0 | 0 | 2 | 0 | 0 | 0 | 0 | 0 | 0 | 0 | 0 | 0 | 0 | 0 | 0 | 0 | 0 | 0 | 0 | 0 |
| Physa | 0 | 0 | 0 | 0 | 0 | 0 | 0 | 0 | 0 | 0 | 0 | 0 | 2 | 0 | 0 | 0 | 0 | 0 | 0 | 0 | 0 | 0 | 0 | 0 | 1 | 0 | 0 | 0 | 0 | 1 |
| Ancylus | 0 | 0 | 0 | 0 | 0 | 0 | 0 | 0 | 0 | 0 | 0 | 0 | 2 | 0 | 0 | 0 | 0 | 0 | 0 | 0 | 0 | 0 | 0 | 0 | 0 | 0 | 0 | 0 | 0 | 1 |
| Amphibola | 0 | 0 | 0 | 0 | 0 | 0 | 0 | 0 | 0 | 0 | 0 | 0 | 2 | 0 | 0 | 0 | 0 | 0 | 0 | 0 | 0 | 0 | 0 | 0 | ? | 0 | 0 | 0 | 0 | 1 |
| Cepaea | 0 | 0 | 1 | 0 | 0 | 0 | 0 | 0 | 0 | 0 | 0 | 0 | 2 | 1 | 1 | 0 | 0 | 0 | 0 | 0 | 0 | 0 | 0 | 0 | 0 | 0 | 0 | 0 | 0 | 0 |
| Limacina | 0 | 0 | 0 | 0 | 0 | 0 | 0 | 0 | 0 | 0 | 0 | 0 | 0 | 0 | 0 | 0 | 0 | 0 | 0 | 0 | 0 | 2 | 0 | 0 | 3 | 1 | 2 | 2 | 1 | 0 |
| Cavolinia | 0 | 0 | 0 | 0 | 0 | 0 | 0 | 0 | 0 | 0 | 0 | 0 | 0 | 0 | 0 | 0 | 0 | 0 | 0 | 0 | 0 | 2 | 0 | 0 | 3 | 1 | 2 | 2 | 1 | 0 |
| Spongiobranchaea | 0 | 0 | 0 | 0 | 0 | 0 | 0 | 0 | 0 | 0 | 0 | 0 | 0 | 0 | 0 | 0 | 0 | 0 | 0 | 0 | 0 | 0 | 0 | 0 | 0 | 0 | 0 | 0 | 0 | 0 |
| Clione | 0 | 0 | 0 | 0 | 0 | 0 | 0 | 0 | 0 | 0 | 0 | 0 | 1 | N | N | 0 | 0 | 0 | 0 | 0 | 0 | 0 | 0 | 0 | 0 | 0 | 0 | 0 | 0 | 0 |

|  |  |  |  |  |  |  |  |  |  |  |  |  |  |  |  |  |  |  |  |  |  |  |  |  |  |  |  |  |  |  |
| --- | --- | --- | --- | --- | --- | --- | --- | --- | --- | --- | --- | --- | --- | --- | --- | --- | --- | --- | --- | --- | --- | --- | --- | --- | --- | --- | --- | --- | --- | --- |
| allzero | 0 | 0 | 0 | 0 | 0 | 0 | 0 | 0 | 0 | 0 | 0 | 0 | 0 | 0 | 0 | 0 | 0 | 0 | 0 | 0 | 0 | 0 | 0 | 0 | 0 | 0 | 0 | 0 | 0 | 0 |
| Valvata | 0 | 0 | 0 | 1 | 1 | 1 | 0 | 0 | 0 | 1 | 1 | 0 | 0 | 1 | 0 | 1 | 1 | 0 | 1 | 0 | 0 | 1 | N | 0 | 0 | 0 | 0 | 0 | 1 | 0 |
| Odostomia* | 0 | 0 | 0 | ? | ? | ? | 0 | 1 | 0 | 1 | ? | 0 | ? | 1 | 0 | 0 | 0 | 1 | ? | 0 | 2 | 2 | 0 | N | 0 | ? | ? | 0 | ? | ? |
| Pyramidella | 0 | 0 | 0 | 0 | 0 | 1 | 0 | 1 | 0 | 1 | ? | 0 | ? | 1 | 0 | 1 | 0 | 1 | 0 | 0 | 2 | 0 | 0 | 0 | 0 | 0 | 0 | 0 | 1 | 0 |
| Heliacus* | 0 | 0 | 0 | 1 | ? | 1 | 0 | 0 | 0 | 1 | ? | 0 | 0 | 1 | 3 | 1 | 2 | 2 | N | 0 | 1 | 0 | 2 | N | 0 | 0 | 0 | 0 | 0 | 0 |
| Gegania* | 0 | 0 | 0 | 1 | ? | 1 | 0 | 0 | 0 | 1 | ? | 0 | 0 | 1 | 3 | 1 | 1 | 2 | N | 0 | 1 | 0 | 2 | N | 0 | ? | 0 | 0 | 0 | 0 |
| Acteon | 0 | 0 | 0 | 1 | 1 | 0 | 0 | 0 | 0 | 1 | 0 | 0 | 0 | 1 | 1 | 1 | 0 | 0 | 1 | 0 | 1 | 3 | N | N | 0 | 1 | 0 | 0 | 1 | 0 |
| Pupa | 0 | 0 | 0 | 1 | 1 | 0 | 0 | 0 | 0 | 0 | 0 | 0 | 0 | 1 | 1 | 1 | 0 | 0 | 1 | 0 | 1 | 3 | N | N | 0 | 1 | 0 | 0 | 1 | 0 |
| Bullina | 0 | 0 | 0 | 0 | 1 | 1 | 2 | 0 | 0 | 1 | ? | 0 | 0 | 1 | 1 | 1 | 0 | 0 | 1 | 0 | 1 | 0 | 0 | 0 | 0 | 1 | 0 | 0 | 1 | ? |
| Micromelo | 0 | 0 | 0 | 0 | 1 | 0 | 2 | 0 | 0 | 1 | ? | 0 | ? | 1 | 1 | 1 | 0 | 0 | 1 | 0 | 1 | 3 | N | N | 0 | 1 | 0 | 0 | 1 | 1 |
| Hydatina | 0 | 0 | 0 | 0 | 1 | 1 | 2 | 0 | 0 | 1 | 0 | 0 | 0 | 1 | 1 | 1 | 0 | 0 | 1 | 0 | 1 | 0 | 0 | 0 | 0 | 1 | 0 | 0 | 0 | 1 |
| Runcina | 2 | 0 | 0 | 0 | 1 | 0 | 2 | 1 | 1 | 1 | ? | 0 | 1 | 1 | 0 | 0 | 1 | 1 | 1 | 1 | 0 | 2 | 0 | N | 0 | 0 | 0 | 0 | 0 | 2 |
| Metaruncina* | 1 | 0 | 0 | 0 | 1 | 0 | 2 | 1 | 1 | 1 | 0 | 0 | 1 | 1 | 0 | 0 | 1 | 1 | 1 | 1 | 0 | 2 | 0 | N | 0 | ? | 1 | 0 | 0 | 2 |
| Ringicula* | 0 | 0 | 0 | 1 | 1 | 1 | ? | 0 | 1 | 0 | ? | ? | ? | 1 | 0 | 0 | 1 | 1 | ? | 1 | 0 | 0 | 0 | 0 | 0 | ? | 0 | 0 | 0 | 0 |
| Diaphana | ? | 0 | 0 | ? | ? | ? | ? | ? | 0 | ? | ? | ? | ? | 1 | ? | 0 | ? | ? | ? | 1 | 0 | ? | ? | ? | 0 | ? | 0 | 0 | 1 | 1 |
| Newnesia | 1 | 0 | 0 | 0 | 0 | 0 | 1 | 0 | 0 | 0 | 1 | 0 | 0 | 1 | 0 | 0 | ? | 1 | 1 | 0 | 0 | 1 | N | 0 | 0 | ? | 0 | 0 | 1 | 1 |
| Colpodaspis | 0 | 0 | 0 | 1 | 0 | 1 | 2 | 0 | 0 | 1 | ? | 0 | 1 | 1 | 0 | 0 | 0 | 1 | 1 | 1 | 0 | 0 | 0 | 0 | 0 | 0 | 0 | 0 | 0 | 1 |
| Bulla | 0 | 0 | 0 | 0 | 0 | 0 | 2 | 0 | 0 | 0 | 1 | 0 | 0 | 1 | 0 | 0 | 0 | 1 | 1 | 1 | 0 | 0 | 1 | 0 | 0 | ? | 0 | 0 | 0 | 2 |
| Haminoea | 2 | 0 | 0 | 0 | 0 | 1 | 2 | 0 | 1 | 0 | 1 | 0 | 0 | 1 | 0 | 0 | 0 | 1 | 1 | 1 | 0 | 0 | 0 | 0 | 0 | 0 | 0 | 0 | 0 | 1 |
| Phanerophthalmus | 2 | 0 | 0 | 0 | 0 | 0 | 2 | 0 | 1 | 1 | 1 | 0 | ? | 1 | 0 | 0 | ? | 1 | 1 | 1 | 0 | 0 | 0 | 0 | 0 | ? | 1 | 0 | 0 | 2 |
| Smaragdinella | 2 | 0 | 0 | 0 | 0 | ? | 2 | 0 | 1 | 0 | ? | 0 | 0 | 1 | 0 | 0 | 0 | 1 | 1 | 1 | 0 | 0 | 0 | 0 | 0 | 0 | 0 | 0 | 0 | 1 |
| Siphopteron | 0 | 0 | 0 | 0 | 1 | 1 | 0 | 1 | 0 | 1 | ? | 0 | 0 | 1 | 0 | 0 | 1 | 1 | 1 | 1 | 0 | 0 | 0 | 0 | 0 | 1 | 0 | 0 | 0 | 1 |
| Sagaminopteron | 0 | 0 | 0 | 0 | 1 | 1 | 0 | 1 | 0 | 1 | ? | 0 | 0 | 1 | 0 | 0 | 1 | 1 | 1 | 1 | 0 | 0 | 0 | 0 | 0 | 1 | 0 | 0 | 0 | 1 |
| Gastropteron | 0 | 0 | 0 | 0 | 0 | 1 | 0 | 1 | 0 | ? | 1 | 0 | 0 | 1 | 0 | 0 | 0 | 1 | 1 | 1 | 0 | ? | ? | 0 | 0 | ? | 0 | 0 | 0 | 1 |
| Philinopsis | 0 | 0 | 0 | 0 | 0 | 0 | 2 | 0 | 0 | 0 | 0 | 0 | 0 | 1 | 0 | 0 | 0 | 1 | 0 | 1 | 0 | 0 | 1 | 0 | 0 | 0 | 0 | 0 | 0 | 1 |
| Chelidonura | 0 | 0 | 0 | 0 | 0 | 0 | 2 | 0 | 0 | 0 | 0 | 0 | 0 | 1 | 0 | 0 | 0 | 1 | 0 | 1 | 0 | 0 | 1 | 0 | 0 | 0 | 0 | 0 | 0 | 1 |
| Philine | 0 | 0 | 0 | 0 | 0 | 1 | 2 | 0 | 0 | 0 | 1 | 0 | 0 | 1 | 0 | 0 | 0 | 1 | 1 | 1 | 0 | 0 | 0 | 0 | 0 | 0 | 0 | 0 | 0 | 1 |
| Cylichna | 0 | 0 | 0 | 0 | 0 | 0 | 2 | 0 | 0 | 0 | 0 | 0 | 0 | 1 | 0 | 0 | 1 | 1 | 0 | 1 | 0 | 0 | 0 | 0 | 0 | 0 | 0 | 0 | 1 | 1 |
| Retusa | ? | ? | 0 | ? | 0 | 0 | ? | ? | 0 | 0 | ? | ? | 0 | 1 | 0 | 0 | 0 | 2 | N | ? | 0 | 0 | 0 | 0 | 0 | ? | 0 | 0 | ? | 1 |
| Scaphander | 0 | 0 | 0 | 0 | 0 | 0 | 2 | 0 | 0 | 0 | 1 | 0 | 0 | 1 | 0 | 0 | 0 | 1 | 1 | 1 | 0 | 0 | 0 | 0 | 0 | 0 | 0 | 0 | 0 | 1 |
| Acteocina | 0 | 0 | 0 | 0 | 0 | 1 | ? | 0 | 0 | 0 | ? | ? | ? | 1 | 0 | 0 | 0 | 1 | 1 | 1 | 0 | 0 | 0 | 0 | 0 | 0 | ? | 0 | ? | ? |
| Akera | 0 | 2 | 1 | 0 | 0 | ? | 1 | 0 | 1 | 1 | 1 | 0 | 0 | 1 | 0 | 0 | 0 | 1 | 1 | 0 | 0 | 0 | 0 | 0 | 1 | 0 | 0 | 0 | 0 | 1 |
| Aplysia | 0 | 2 | 1 | 0 | 0 | ? | 1 | 0 | 1 | 1 | 1 | 0 | 0 | 1 | 0 | 0 | 0 | 1 | 1 | 0 | 0 | 0 | 0 | 0 | 1 | 0 | 0 | 0 | 0 | 1 |
| Dolabella | 0 | 2 | 1 | 0 | 0 | ? | 1 | 0 | 1 | 1 | 1 | 0 | 0 | 1 | 0 | 0 | 0 | 1 | 1 | 0 | 0 | 0 | 0 | 0 | 1 | ? | 0 | 0 | 0 | 1 |
| Cylindrobulla* | 1 | 0 | 0 | 0 | 0 | 2 | ? | 0 | 1 | 0 | ? | ? | ? | 1 | 1 | 4 | 0 | 1 | 1 | 0 | 0 | 0 | 2 | N | 0 | ? | 0 | 0 | 0 | ? |
| Ascobulla* | 1 | 0 | 0 | ? | 0 | 1 | 1 | 0 | 1 | 0 | ? | ? | ? | 1 | 1 | 4 | 0 | 1 | 1 | ? | 0 | 0 | 2 | N | 0 | ? | ? | 0 | ? | 2 |
| Elysia | 1 | 0 | 0 | 0 | 1 | 1 | 2 | 1 | 1 | 1 | 0 | 0 | 1 | 1 | 1 | 1 | 0 | 1 | 1 | 0 | 0 | 1 | N | 0 | 0 | 0 | 0 | 0 | N | 2 |
| Oxynoe | 1 | 0 | 0 | 0 | ? | 1 | 2 | 1 | 1 | 1 | ? | ? | 1 | 1 | 1 | 1 | 0 | 1 | 1 | ? | 0 | 1 | N | 0 | 0 | 0 | 0 | 0 | 0 | 2 |
| Lobiger | 1 | 0 | 0 | ? | 0 | ? | 2 | 1 | 1 | 1 | 0 | ? | 1 | 1 | 1 | 0 | 0 | 1 | 1 | ? | 0 | 0 | 1 | 0 | 0 | ? | 0 | 0 | 0 | 2 |
| Tylodina | 0 | 0 | 0 | 0 | 0 | 1 | 2 | 1 | 1 | 1 | 1 | 0 | 0 | 1 | 0 | 1 | 0 | 0 | 0 | 0 | 2 | 0 | 2 | N | 0 | 0 | 0 | 0 | 0 | ? |
| Umbraculum | 0 | 0 | 0 | 0 | 0 | 1 | 2 | 1 | 1 | 1 | 0 | 0 | 0 | 1 | 0 | 1 | 0 | 0 | 0 | 0 | 2 | 0 | 2 | N | 0 | 0 | 0 | 0 | 0 | 1 |
| Hedylopsis* | 0 | 0 | 0 | 0 | 0 | ? | 2 | 1 | 0 | 1 | ? | 0 | 1 | 1 | 0 | 0 | 2 | 1 | 1 | 1 | 0 | 3 | N | N | 0 | 0 | 0 | 0 | N | 2 |
| Microhedyle* | 0 | 0 | 0 | 0 | 0 | ? | ? | ? | 0 | 1 | ? | ? | 1 | 0 | N | 0 | 2 | 2 | N | N | 0 | 3 | N | N | 0 | 0 | 0 | 0 | N | ? |
| Unela* | 0 | 0 | 0 | 0 | 0 | ? | 2 | 1 | 0 | 1 | ? | 0 | 1 | 0 | N | 0 | 2 | 2 | N | N | 0 | 3 | N | N | 0 | 0 | 0 | 0 | N | N |
| Philinoglossa* | 0 | 0 | 0 | 0 | 0 | 1 | 2 | 1 | 0 | 1 | ? | 0 | 1 | 1 | 0 | 0 | 1 | 2 | N | 1 | 0 | 3 | N | N | 0 | ? | 1 | 0 | N | 3 |
| Pluscula* | 0 | 0 | 0 | 0 | 0 | 1 | 2 | 1 | 0 | 1 | ? | 0 | 1 | 1 | 0 | 0 | 0 | 1 | 1 | 1 | ? | 1 | N | 0 | 0 | ? | 1 | 0 | N | 3 |
| Philinoglossa_nov | 0 | 0 | 0 | 0 | 0 | 1 | 2 | ? | 0 | 1 | ? | ? | 1 | 1 | ? | 0 | 1 | 2 | N | 1 | 0 | 1 | N | 0 | 0 | 0 | ? | 0 | N | ? |
| Tomthompsonia | 0 | 0 | 0 | 0 | 1 | 1 | 0 | 1 | 0 | 1 | ? | 0 | 0 | 1 | 1 | 1 | 1 | 1 | 1 | 1 | 1 | 0 | 2 | N | 0 | 0 | 0 | 0 | 0 | 1 |
| Berthella | 0 | 0 | 0 | 0 | 1 | 1 | 0 | 1 | 0 | 1 | 0 | 0 | 0 | 1 | 1 | 1 | 0 | 1 | 1 | 1 | 1 | 0 | 2 | N | 0 | 0 | 0 | 0 | 0 | 1 |
| Bathyberthella | 0 | 0 | 0 | 0 | 1 | 1 | 0 | 1 | 0 | 1 | 0 | 0 | 0 | 1 | 2 | 1 | ? | 1 | 1 | 0 | 1 | 0 | 0 | 0 | 0 | ? | 0 | 0 | 0 | 1 |
| Bathydoris | 0 | 1 | 0 | 0 | 0 | ? | 2 | 1 | 1 | 1 | 0 | 1 | ? | 1 | 1 | 1 | 1 | 1 | 1 | 0 | 1 | 1 | N | 0 | 0 | 0 | 1 | 0 | 0 | 2 |
| Goniodoris | 0 | 0 | 0 | 0 | 1 | 0 | 2 | 1 | 1 | 1 | 0 | 1 | 0 | 1 | 2 | 1 | 0 | 1 | 1 | 0 | 1 | 0 | 0 | 0 | 0 | 0 | 1 | 0 | 0 | 2 |
| Jorunna | 0 | 1 | 0 | 0 | 1 | 1 | 2 | 1 | 1 | 1 | ? | 1 | 0 | 1 | 2 | 1 | 0 | 1 | 1 | 0 | 1 | 0 | 0 | 0 | 0 | 0 | 1 | 0 | 0 | 2 |
| Austrodoris | 0 | 1 | 0 | 0 | 1 | 1 | 2 | 1 | 1 | 1 | 0 | 1 | 0 | 1 | 2 | 1 | ? | 1 | 1 | 0 | 1 | 0 | 0 | 0 | 0 | 0 | 1 | 0 | 0 | 2 |
| Polycera | 0 | 1 | 0 | 0 | 1 | 1 | 2 | 1 | 1 | 1 | 0 | ? | 0 | 1 | 2 | 1 | 1 | 1 | 1 | 0 | 1 | 0 | 0 | 0 | 0 | 0 | 1 | 0 | 0 | 2 |
| Limacia | 0 | 1 | 0 | 0 | 1 | 1 | 2 | 1 | 1 | 1 | ? | ? | 0 | 1 | 2 | 1 | ? | 1 | 1 | 0 | 1 | 0 | 0 | 0 | 0 | ? | 1 | 0 | 0 | 2 |
| Rhodope | 0 | 0 | 0 | 0 | 0 | ? | 2 | 1 | 1 | 1 | ? | 0 | 1 | 1 | 0 | 1 | 1 | ? | 0 | 0 | 1 | 1 | N | 1 | 0 | ? | N | 0 | N | N |
| Helminthope | 0 | 0 | 0 | 0 | ? | ? | 2 | 0 | 1 | 1 | ? | ? | 1 | 1 | 0 | 1 | 1 | 2 | N | 0 | ? | 1 | N | 1 | 0 | ? | N | 0 | N | N |
| Doridoxa | 0 | 0 | 0 | 0 | ? | 0 | 2 | ? | 1 | 1 | 0 | ? | 0 | 1 | 1 | 1 | 1 | 1 | 1 | 0 | 1 | 0 | ? | ? | 0 | ? | 0 | 0 | N | 2 |
| Armina | 0 | 0 | 0 | 0 | 1 | 0 | 2 | 1 | 1 | 1 | 1 | 1 | 0 | 1 | 1 | 1 | 0 | 1 | 1 | 0 | 1 | 2 | 1 | N | 0 | 0 | 0 | 0 | N | 2 |
| Dermatobranchus | 0 | 0 | 0 | 0 | 1 | 0 | 2 | 1 | 1 | 1 | 1 | 1 | 0 | 1 | 1 | 1 | 0 | 1 | 1 | 0 | 1 | 2 | 1 | N | 0 | 0 | 0 | 0 | N | 2 |
| Dendronotus | 0 | 0 | 0 | 1 | 1 | 0 | 2 | 1 | 1 | 1 | 0 | 1 | 0 | 1 | 2 | 1 | 1 | 1 | 1 | 0 | 1 | 0 | 0 | 0 | 0 | 1 | 0 | 0 | N | 2 |
| Tritonia | 0 | 0 | 0 | 1 | 1 | 0 | 2 | 1 | 1 | 1 | 1 | 1 | 0 | 1 | 1 | 1 | 1 | 1 | 1 | 0 | 1 | 2 | 1 | N | 0 | 0 | 0 | 0 | N | 2 |
| Pseudotritonia | 0 | 0 | 0 | 0 | 1 | 0 | 2 | 1 | 1 | 1 | 0 | 1 | ? | 1 | 1 | 1 | 0 | 1 | 1 | 0 | 1 | 2 | 1 | N | 0 | 0 | 0 | 0 | N | 2 |
| Charcotia | 0 | 0 | 0 | 0 | 1 | 1 | 2 | 1 | 1 | 1 | 0 | 1 | 0 | 1 | 1 | 1 | 0 | 1 | 1 | 0 | 1 | 2 | 1 | N | 0 | 0 | 0 | 0 | N | 2 |
| Notaeolidia | 0 | 0 | 0 | 0 | 1 | 0 | 2 | 1 | 1 | 1 | 1 | 1 | 0 | 1 | 1 | 1 | 0 | 1 | 1 | 0 | 1 | 2 | 1 | N | 0 | 1 | 0 | 0 | N | 2 |
| Flabellina | 0 | 0 | 0 | 0 | 1 | 0 | 2 | 1 | 1 | 1 | 0 | 1 | 0 | 1 | 1 | 1 | 0 | 1 | 1 | 0 | 1 | 2 | 1 | N | 0 | 1 | 0 | 0 | N | 2 |
| Aeolidia | 0 | 0 | 0 | 0 | 1 | 0 | 2 | 1 | 1 | 1 | 0 | 1 | 0 | 1 | 1 | 1 | 0 | 1 | 1 | 0 | 1 | 2 | 1 | N | 0 | 1 | 0 | 0 | N | 2 |
| Onchidium | 0 | 0 | 0 | 1 | 0 | 0 | 2 | 1 | 1 | 0 | 0 | 0 | 0 | 1 | 0 | 3 | ? | 1 | 1 | 1 | 0 | 0 | 0 | 0 | ? | ? | 1 | 1 | N | 4 |
| Onchidella | 0 | 0 | 0 | 1 | 0 | 0 | 2 | 1 | 1 | 0 | 0 | 0 | ? | 1 | 0 | 3 | ? | 1 | 1 | 0 | 0 | 0 | 0 | 0 | ? | 0 | 1 | 1 | N | 2 |
| Siphonaria | 0 | 0 | 0 | 0 | 0 | 1 | 2 | 1 | 1 | 0 | ? | 0 | 0 | 1 | 0 | 1 | 1 | 1 | 1 | 1 | 1 | 0 | 0 | 0 | 0 | 0 | 0 | 1 | 1 | 1 |
| Ellobiidae | 0 | ? | 0 | 0 | 1 | ? | 1 | 0 | 1 | 1 | 1 | 0 | 0 | 1 | 0 | 3 | 1 | 1 | 1 | 0 | 0 | 1 | N | 0 | 0 | 1 | 0 | 1 | N | 1 |
| Physa | 0 | 1 | 0 | 0 | 0 | 1 | 2 | 1 | 1 | 1 | ? | 0 | 0 | 1 | 1 | 1 | 0 | 1 | 1 | 0 | 0 | 0 | 0 | 0 | 0 | 1 | 0 | 1 | N | 1 |
| Ancylus | 0 | 0 | 0 | 1 | 0 | 1 | 2 | 1 | 1 | 1 | ? | 0 | 0 | 1 | 0 | 1 | 0 | 1 | 1 | 1 | 0 | 1 | N | 0 | 0 | 1 | 0 | 0 | N | 3 |
| Amphibola | 0 | ? | 0 | 0 | 0 | 1 | 1 | 1 | 0 | 0 | 1 | 0 | ? | 1 | 0 | 2 | ? | 1 | 0 | 1 | 1 | 2 | 0 | N | 0 | ? | 0 | 1 | N | 0 |
| Cepaea | 0 | 0 | 0 | 0 | 0 | ? | 2 | 1 | 1 | 1 | ? | 0 | ? | 1 | 0 | 1 | ? | 1 | 1 | 0 | 1 | 1 | N | 0 | 0 | ? | ? | 1 | N | 0 |
| Limacina | 0 | 2 | 0 | 0 | 0 | 2 | 2 | 1 | 1 | 0 | ? | 0 | 0 | 1 | 0 | 0 | 0 | 1 | 1 | 1 | 0 | 0 | 1 | 0 | 0 | ? | 0 | 0 | N | 0 |
| Cavolinia | 0 | 2 | 0 | 0 | 0 | 2 | 2 | 1 | 1 | 0 | ? | 0 | 0 | 1 | 0 | 0 | 1 | ? | 1 | 0 | 0 | 2 | 1 | N | 0 | ? | 1 | 0 | 0 | 2 |
| Spongiobranchaea | 0 | 0 | 0 | 0 | 0 | 1 | 1 | 1 | 1 | 0 | 1 | 0 | 0 | 1 | 0 | 0 | ? | 1 | 0 | 0 | 0 | 2 | 1 | N | 0 | ? | 1 | 0 | N | 2 |
| Clione | 0 | 0 | 0 | 0 | 0 | 0 | 1 | 1 | 1 | 0 | 1 | 0 | 0 | 1 | 0 | 0 | ? | 1 | 0 | 0 | 0 | 2 | 1 | N | 0 | ? | 1 | 0 | N | 2 |

|  |  |  |  |  |  |  |  |  |  |  |  |  |  |  |  |  |  |  |  |  |
| --- | --- | --- | --- | --- | --- | --- | --- | --- | --- | --- | --- | --- | --- | --- | --- | --- | --- | --- | --- | --- |
| allzero | 0 | 0 | 0 | 0 | 0 | 0 | 0 | 0 | 0 | 0 | 0 | 0 | 0 | 0 | 0 | 0 | 0 | 0 | 0 | 0 |
| Valvata | 0 | 0 | 0 | 0 | 0 | 1 | 0 | 0 | 0 | 0 | 0 | 0 | 0 | 0 | 0 | 0 | 0 | ? | 0 | 0 |
| Odostomia* | ? | ? | ? | 0 | 0 | 0 | ? | 0 | 0 | 0 | 0 | 0 | ? | 0 | 0 | 0 | 0 | 1 | 0 | ? |
| Pyramidella | 0 | 0 | 0 | 0 | 0 | 1 | ? | 0 | 0 | 0 | 0 | 0 | ? | 0 | 0 | 0 | ? | 1 | 0 | ? |
| Heliacus* | 0 | 0 | 0 | 0 | 0 | 0 | ? | 0 | 0 | 0 | 0 | 0 | ? | 0 | 0 | 0 | 0 | 0 | 0 | ? |
| Gegania* | 0 | 0 | 0 | 0 | 0 | 0 | ? | 0 | 0 | 0 | 0 | 0 | ? | 0 | 0 | 0 | ? | ? | ? | ? |
| Acteon | 1 | 0 | 0 | 0 | 0 | 0 | 0 | 0 | 0 | 0 | 0 | 0 | 0 | 0 | 0 | 0 | 0 | 1 | 0 | 0 |
| Pupa | 1 | 0 | 0 | 0 | 0 | 0 | 0 | 0 | 0 | 0 | 0 | 0 | ? | 0 | 0 | 0 | 0 | ? | ? | 0 |
| Bullina | 1 | 0 | 0 | 0 | 0 | 0 | 0 | 0 | 0 | 0 | 0 | 0 | ? | 0 | 0 | 0 | 0 | ? | ? | 0 |
| Micromelo | 1 | 0 | 0 | 0 | 0 | 0 | ? | 0 | 0 | 0 | 0 | 0 | ? | 0 | 0 | 0 | 0 | ? | ? | 0 |
| Hydatina | 1 | 0 | ? | 0 | 0 | 0 | ? | 0 | 0 | 0 | 0 | 0 | 0 | 0 | 0 | 0 | 0 | ? | ? | 0 |
| Runcina | 0 | 0 | 0 | 0 | 0 | 0 | 0 | 0 | 1 | 0 | 0 | 0 | 1 | 0 | 0 | 0 | 0 | 1 | 0 | 0 |
| Metaruncina* | 0 | 0 | 0 | 0 | 0 | 0 | ? | 0 | 1 | 0 | 1 | 0 | ? | 0 | 0 | 0 | 0 | ? | ? | ? |
| Ringicula* | ? | ? | ? | 0 | 0 | 0 | ? | 0 | 0 | 0 | 0 | 0 | ? | ? | 0 | 0 | 0 | ? | ? | ? |
| Diaphana | ? | ? | ? | 0 | 0 | 0 | ? | 0 | 0 | 0 | 0 | 0 | ? | ? | 0 | 0 | 0 | 1 | 0 | ? |
| Newnesia | ? | ? | 0 | 0 | 0 | 0 | 0 | 0 | 0 | 0 | 0 | 0 | 0 | 0 | 0 | 0 | 0 | ? | ? | 1 |
| Colpodaspis | 0 | 0 | 0 | 0 | 0 | 0 | ? | 0 | 0 | 0 | 0 | 0 | 0 | 0 | 0 | 0 | 0 | ? | ? | ? |
| Bulla | 0 | 0 | 0 | 0 | 0 | 0 | 1 | 0 | 0 | 0 | 0 | 0 | 0 | 0 | 0 | 0 | 0 | 1 | 0 | ? |
| Haminoea | 1 | 0 | 0 | 0 | 0 | 0 | 1 | 0 | 0 | 0 | 0 | 0 | 0 | 0 | 0 | 0 | 0 | 1 | 0 | 0 |
| Phanerophthalmus | 1 | 0 | 0 | 0 | 0 | 0 | 0 | 0 | 0 | 0 | 0 | 0 | ? | 0 | 0 | 0 | 0 | ? | ? | 1 |
| Smaragdinella | ? | ? | 0 | 0 | 0 | 0 | 0 | 0 | 0 | 0 | 0 | 0 | ? | 0 | 0 | 0 | 0 | ? | ? | 1 |
| Siphopteron | 1 | 0 | 0 | 0 | 0 | 1 | 0 | 0 | 0 | 0 | 0 | 0 | ? | 0 | 0 | 0 | 0 | ? | ? | 1 |
| Sagaminopteron | 1 | 0 | 0 | 0 | 0 | 1 | 0 | 0 | 0 | 0 | 0 | 0 | ? | 0 | 0 | 0 | 0 | ? | ? | 1 |
| Gastropteron | 1 | 0 | 0 | 0 | 0 | ? | ? | 0 | 0 | 0 | 0 | 0 | 0 | 0 | 0 | 0 | 0 | 1 | 0 | ? |
| Philinopsis | 1 | 0 | 0 | 0 | 0 | 0 | 0 | 0 | 0 | 0 | 0 | 0 | 0 | 0 | 1 | 0 | 0 | ? | ? | 1 |
| Chelidonura | 1 | 0 | 0 | 0 | 0 | 0 | ? | 0 | 0 | 0 | 0 | 0 | 0 | 0 | 1 | 0 | 0 | ? | ? | 1 |
| Philine | 1 | 0 | 0 | 0 | 0 | 1 | ? | 0 | 0 | 0 | 0 | 0 | 0 | 0 | 1 | 0 | 0 | 1 | 0 | ? |
| Cylichna | 0 | 0 | 0 | 0 | 0 | 0 | 1 | 0 | 0 | 0 | 0 | 0 | 0 | 0 | 0 | 0 | 0 | ? | ? | ? |
| Retusa | ? | ? | 0 | 0 | 0 | ? | ? | ? | 0 | 0 | 0 | 0 | ? | ? | 0 | 0 | 0 | 1 | 0 | ? |
| Scaphander | ? | ? | 0 | 0 | 0 | 0 | 1 | 0 | 0 | 0 | 0 | 0 | 0 | 0 | 0 | 0 | 0 | 1 | 0 | ? |
| Acteocina | ? | ? | 0 | 0 | 0 | 0 | 0 | 0 | 0 | 0 | 0 | 0 | ? | 0 | 0 | 0 | 0 | ? | ? | ? |
| Akera | 0 | 0 | 0 | 0 | 0 | 0 | 1 | 1 | 0 | 0 | 0 | 0 | 0 | 0 | 0 | 0 | 0 | 1 | 0 | 1 |
| Aplysia | 0 | 0 | 0 | 0 | 0 | 1 | 1 | 1 | 0 | 0 | 0 | 0 | ? | 0 | 0 | 0 | 0 | 1 | 0 | 1 |
| Dolabella | 0 | 0 | 0 | 0 | 0 | 1 | 1 | 1 | 0 | 0 | 0 | 0 | ? | 0 | 0 | 0 | 0 | 1 | 0 | 1 |
| Cylindrobulla* | ? | ? | 0 | 0 | 0 | 0 | ? | 0 | 0 | 0 | 0 | 0 | 0 | ? | 0 | 0 | 0 | ? | ? | ? |
| Ascobulla* | ? | ? | 0 | 0 | 0 | 0 | ? | 0 | 0 | 0 | 0 | 0 | 0 | ? | 0 | 0 | 0 | ? | ? | ? |
| Elysia | 0 | 0 | 0 | 0 | 0 | 0 | 0 | 0 | 0 | 0 | 0 | 0 | 1 | 0 | N | 0 | 0 | 1 | 0 | 1 |
| Oxynoe | 0 | 0 | 0 | 0 | 0 | 0 | 0 | 0 | 0 | 0 | 0 | 0 | 0 | 0 | 0 | 0 | 0 | ? | ? | 0 |
| Lobiger | 0 | 0 | ? | 0 | 0 | 0 | ? | 0 | 0 | 0 | 0 | 0 | ? | ? | ? | 0 | 0 | ? | ? | ? |
| Tylodina | 0 | 0 | 0 | 0 | 0 | 1 | 0 | 0 | 0 | 0 | 0 | 0 | 0 | 0 | 0 | 0 | 0 | ? | ? | 0 |
| Umbraculum | 0 | 0 | 0 | 0 | 0 | 1 | 0 | 0 | 0 | 0 | 0 | 0 | 1 | 0 | 0 | 0 | 0 | 1 | 1 | 0 |
| Hedylopsis* | 0 | 0 | 0 | 0 | 0 | ? | ? | ? | 0 | 0 | 0 | 0 | 0 | 1 | 0 | 0 | ? | 1 | 1 | ? |
| Microhedyle* | 0 | 0 | 0 | 0 | 0 | ? | ? | ? | 0 | 0 | 0 | 0 | 0 | 1 | 0 | 0 | ? | ? | ? | ? |
| Unela* | 0 | 0 | 0 | 0 | 0 | 0 | 0 | 0 | 0 | 0 | 0 | 0 | 0 | 1 | 0 | 0 | ? | 1 | 1 | ? |
| Philinoglossa* | 0 | 0 | 0 | 0 | 0 | ? | 0 | 0 | 0 | 0 | 0 | ? | 0 | 0 | 0 | 0 | ? | 1 | 1 | ? |
| Pluscula* | 0 | 0 | 0 | 0 | 0 | ? | 0 | 0 | 0 | 0 | 0 | ? | ? | 0 | 0 | 0 | ? | ? | ? | ? |
| Philinoglossa_nov | 0 | 0 | 0 | 0 | 0 | 0 | 0 | 0 | 0 | 0 | 0 | 0 | ? | 0 | 0 | 0 | ? | ? | ? | ? |
| Tomthompsonia | 1 | 0 | 1 | 0 | 1 | 1 | 0 | 0 | 0 | 0 | 0 | 0 | 1 | 1 | 0 | 0 | 0 | 1 | ? | 0 |
| Berthella | 1 | 0 | 1 | 0 | 1 | 1 | 0 | 0 | 0 | 0 | 0 | 0 | 1 | 1 | 0 | 0 | 0 | 1 | ? | 0 |
| Bathyberthella | 1 | 0 | 1 | 0 | 1 | 1 | 0 | 0 | 0 | 0 | 0 | 0 | 1 | 1 | 0 | 0 | 0 | 1 | ? | 0 |
| Bathydoris | 1 | 0 | 1 | 0 | 0 | 1 | 0 | 0 | 0 | 0 | 0 | 1 | 1 | 0 | 0 | 0 | 0 | 1 | ? | 0 |
| Goniodoris | 1 | 0 | 1 | 0 | 0 | 1 | 0 | 0 | 0 | 0 | 1 | 1 | 1 | 1 | 0 | 0 | 0 | 1 | ? | 0 |
| Jorunna | 1 | 1 | 1 | 0 | 0 | 1 | 0 | 0 | 0 | 0 | 1 | 1 | 1 | 1 | 0 | 0 | 0 | 1 | ? | 0 |
| Austrodoris | 1 | 1 | 1 | 0 | 0 | 1 | 0 | 0 | 0 | 0 | 1 | 1 | 1 | 1 | 0 | 0 | 0 | 1 | ? | 0 |
| Polycera | 1 | 1 | 1 | 0 | 0 | 1 | 0 | 0 | 0 | 0 | 1 | 1 | 1 | 1 | 0 | 0 | 0 | 1 | ? | 0 |
| Limacia | 1 | 1 | 1 | 0 | 0 | 1 | 0 | 0 | 0 | 0 | 0 | 0 | 1 | 1 | 0 | 0 | 0 | 1 | ? | 0 |
| Rhodope | 0 | 0 | N | 0 | 0 | 0 | 0 | 0 | 0 | 0 | 0 | 0 | ? | 1 | 0 | 0 | 0 | 1 | 1 | ? |
| Helminthope | 0 | 0 | N | 0 | 0 | 0 | 0 | 0 | 0 | 0 | 0 | 0 | ? | 1 | 0 | 0 | 0 | ? | ? | ? |
| Doridoxa | 1 | 0 | 1 | 0 | 0 | 1 | 0 | 0 | 0 | 0 | 0 | 1 | 1 | 0 | 0 | 0 | 0 | ? | ? | ? |
| Armina | 0 | 0 | 1 | 0 | 0 | 1 | 0 | 0 | 0 | 1 | 0 | 1 | 1 | 0 | 0 | 0 | 0 | 1 | 1 | 0 |
| Dermatobranchus | 0 | 0 | 1 | 0 | 0 | 1 | 0 | 0 | 0 | 1 | 0 | 1 | 1 | 0 | 0 | 0 | 0 | 1 | ? | 0 |
| Dendronotus | 0 | 0 | 1 | 0 | 0 | 1 | 0 | 0 | 0 | 0 | 0 | 1 | 1 | 0 | 0 | 0 | 0 | 1 | ? | 0 |
| Tritonia | 0 | 0 | 1 | 0 | 0 | 1 | 0 | 0 | 0 | 0 | 0 | 1 | 1 | 0 | 0 | 0 | 0 | 1 | 1 | 0 |
| Pseudotritonia | 0 | 0 | 1 | 0 | 0 | 1 | 0 | 0 | 0 | 0 | 0 | 1 | 1 | 0 | 0 | 0 | 0 | 1 | ? | 1 |
| Charcotia | 0 | 0 | 1 | 0 | 0 | 1 | 0 | 0 | 0 | 0 | 0 | 1 | 1 | 0 | 0 | 0 | 0 | 1 | ? | 1 |
| Notaeolidia | 0 | 0 | 1 | 1 | 0 | 1 | 0 | 0 | 0 | 0 | 0 | 1 | 1 | 0 | 0 | 0 | 0 | 1 | ? | 1 |
| Flabellina | 0 | 0 | 1 | 1 | 0 | 1 | 0 | 0 | 0 | 0 | 0 | 1 | 1 | 0 | 0 | 0 | 0 | 1 | ? | 1 |
| Aeolidia | 0 | 0 | 1 | 1 | 0 | 1 | 0 | 0 | 0 | 0 | 0 | 1 | 1 | 0 | 0 | 0 | 0 | 1 | 1 | 0 |
| Onchidium | 0 | 0 | 0 | 0 | 0 | 1 | 0 | 0 | 0 | 0 | 0 | 0 | 1 | 0 | 0 | 0 | 1 | 0 | 0 | 0 |
| Onchidella | 0 | 0 | 0 | 0 | 0 | 1 | 0 | 0 | 0 | 0 | 0 | 0 | 1 | 0 | 0 | 0 | 1 | 0 | 0 | 0 |
| Siphonaria | 0 | 0 | 0 | 0 | 0 | 1 | 0 | 0 | 0 | 0 | 0 | 0 | 0 | 0 | 0 | 0 | 1 | 0 | 0 | 0 |
| Ellobiidae | 0 | 0 | 0 | 0 | 0 | 0 | 0 | 0 | 0 | 0 | 0 | 0 | ? | 0 | 0 | 0 | 1 | 0 | 0 | 0 |
| Physa | 1 | 0 | 0 | 0 | 0 | 1 | 0 | 0 | 0 | 0 | 0 | 0 | ? | 0 | 0 | 0 | 1 | 0 | ? | 0 |
| Ancylus | 0 | 0 | 0 | 0 | 0 | 1 | 0 | 0 | 0 | 0 | 0 | 0 | ? | 0 | 0 | 0 | 1 | 0 | ? | 0 |
| Amphibola | 0 | 0 | ? | 0 | 0 | 0 | 0 | 0 | 0 | 0 | 0 | 0 | ? | 0 | 0 | 0 | 1 | 0 | ? | ? |
| Cepaea | 0 | 0 | ? | 0 | 0 | 1 | 0 | 0 | 0 | 0 | 0 | 0 | ? | 0 | 0 | 0 | 1 | 0 | ? | 0 |
| Limacina | 0 | 0 | 0 | 0 | 0 | 0 | 0 | 0 | 0 | 0 | 0 | 0 | 0 | 0 | 0 | 0 | 0 | ? | 0 | 0 |
| Cavolinia | 0 | 0 | 0 | 0 | 0 | 1 | 0 | 0 | 0 | 0 | 0 | 0 | 0 | 0 | 0 | 0 | 0 | ? | 0 | 0 |
| Spongiobranchaea | 0 | 0 | 0 | 0 | 0 | 1 | 1 | 0 | 0 | 0 | 0 | 0 | 0 | 0 | 0 | 1 | 0 | 0 | 0 | 0 |
| Clione | 0 | 0 | 0 | 0 | 0 | 1 | 1 | 0 | 0 | 0 | 0 | 0 | 0 | 0 | 0 | 1 | 0 | 0 | 0 | 0 |
