# Supplementary material for: Opisthobranchia (Mollusca, Gastropoda) – more than just slimy slugs. Shell reduction and its implications on defence and foraging
Source: Front Zool. 2005 Feb 16;2:3. doi: 10.1186/1742-9994-2-3 (PMC554092; doi:10.1186/1742-9994-2-3)
Supplement: Additional File 2 — Characters – Characters and coding of character states used for phylogenetic analysis presented in Figure 3. [file 1742-9994-2-3-S2.doc]

Table 1

|  | Character | Character states |
| --- | --- | --- |
| 1. * | Shell | 0 present and outside, 1 absent, 2 internalized |
|  | Shell shape | 0 coiled (at least 3 coils), 1 circular (limpet-like), 2 less than 3 coils, 3 cavolinid |
|  | Shell spire | 0 well-elevated, 1 sunken |
|  | Operculum (in adult) | 0 present, 1 absent |
|  | Cuticle covering external epidermis | 0 absent, 1 present |
|  | Posterior pallial lobe | 0 absent, 1 present |
|  | Anterior pedal gland | 0 absent, 1 long anterior gland opening between foot and mouth staining bluish, 2 gland in similar position, staining bright red |
|  | Posterior pedal gland | 0 absent, 1 present as a stripe without duct, 2 present with duct |
|  | Parapodia | 0 absent, 1 present, not distinct from foot |
|  | Cephalic shield | 0 absent, 1 present |
|  | Head shield modified into a median unpaired, rolled structure | 0 absent, 1 present |
|  | Cephalic shield, posterior part | 0 continuous in front, posterior with two lobes, 1 divided into two separate lateral lobes, 2 undivided at the posterior rim, N no cephalic shield present |
|  | Rhinophore | 0 absent, 1 present, enrolled, 2 solid and smooth, 3 solid and lamellate |
|  | Cephalic tentacles and connection with eyes | 0 eyes lying at stalked base of rhinophore, 1 eyes present at tip of cephalic tentacle, 2 eyes present and retractile into tentacle, 3 independent from tentacle lying inside, 4 reduced and at tip of tentacle |
|  | Hancock’s organ | 0 absent, 1 present |
|  | Oral tentacular processes | 0 absent, 1 rolled, 2 solid |
|  | Propodial tentacles (foot tentacles) | 0 absent, 1 present |
|  | Sensory bristles on either side of the mouth | 0 absent, 1 present |
|  | Pallial cavity | 0 present without pneumostome, 1 present with pneumostome, 2 absent |
|  | Mantle cavity opening | 0 anterior, 1 lateral, 2 posterior |
|  | Ciliated stripes | 0 present, 1 absent |
|  | Pallial caecum | 0 absent or short, 1 long |
|  | Anterior notum in relation to head | 0 notum free from head, 1 lateral and frontal notal margin fused with head |
|  | Notum in relation to foot (length, not width) | 0 same size, 1 notum larger than foot, 2 notum smaller than foot |
|  | Notal margin | 0 free, 1 fused with foot |
|  | Notal edge | 0 smooth or with branches, 1 papillae containing digestive diverticula |
|  | Anterior edge of head region | 0 smooth, 1 bearing frontal processes |
|  | Gill attachment | 0 to edge of kidney, 1 on surface of kidney |
|  | Plicatidium (gills in mantle cavity) | 0 absent, 1 present and two sided, 2 present and one sided |
|  | Branchial circlet (Doridoidea) | 0 absent, 1 present |
|  | Retractability of anal gills | 0 not retractile, 1 retractile |
|  | Gill foliobranch, a derivation of the hypobranchial gland | 0 absent, 1 present |
|  | Osphradium | 0 present, 1 absent |
|  | Anus | 0 anterior, 1 posterior, median and dorsal, 2 posterior, median in mantle cavity , 3 posterior and ventral, lying externally of mantle cavity |
|  | Evaginable proboscis | 0 absent, 1 present and architectonicid-like , 2 present and pyramidellid-like |
| 1. 2 | Oral cuticle | 0 smooth or absent, 1 with processes, 2 thickened cuticular ring |
|  | Oral glands producing acid mucopolysaccharides | 0 consisting of a layer or absent, 1 distinct glands with two ducts, 2 distinct glands with one duct |
|  | Acid gland opening dorsally in oral tube | 0 absent, 1 present |
|  | Median buccal gland (Hydatinidae) | 0 absent, 1 present |
|  | Buccal gland inside of pharynx | 0 absent, 1 present |
|  | Median buccal pouch | 0 absent, 1 present |
|  | Posterolateral pouches at the pharynx | 0 absent, 1 present |
|  | Jaws | 0 present (laterally), 1 absent, 2 mainly dorsally |
|  | Composition of jaws | 0 composed of platelets, 1 aliform or solid, 2 transformed into stylet |
|  | Masticatory border of aliform jaws | 0 with several rows of denticles, 1 smooth |
|  | Descending ascus | 0 absent, 1 present |
|  | Radula | 0 present, 1 absent |
|  | Rhachidian tooth | 0 present, 1 absent |
|  | Rhachidian tooth | 0 rhomboid with larger median cusp, 1 rhomboid, bilobed cutting edge and median indentation, 2 dagger shaped, 3 elongate plate, 4 comb-like, 5 reduced, but still with small denticle |
|  | Tooth size of rhachidian | 0 uniform, 1 increasing within ribbon |
|  | Lateral teeth | 0 present, 1 absent |
|  | Lateral teeth | 0 several laterals present: First lateral similar in shape and size as all other laterals, but first lateral my also have some denticles, 1 first lateral considerably bigger than all other laterals – the latter hook-shaped, 2 only first lateral present, 3 marginal laterals plate-like, 4 first lateral plate-like, 5 several laterals, large denticles arranged like a comb |
|  | Rodlets at transition of pharynx into oesophagus | 0 absent, 1 present |
|  | Salivary glands | 0 without a bulb at the efferent duct, 1 with a bulb at the efferent duct |
|  | Cuticle in oesophagus | 0 absent, 1 restricted to a small portion within proximal oesophagus, 2 present along whole oesophagus, 3 absent in the proximal part, but starts in the oesophageal crop or gizzard |
|  | Oesophageal gizzard with gizzard plates | 0 plates absent, 1 plates present |
|  | Gizzard plate number | 0 more than four plates, 1 three plates, 2 four plates |
|  | Gizzard plate structure | 0 ridged, 1 tuberculate, 2 smooth, 3 ridged and with spines |
|  | Gizzard spines | 0 absent, 1 present as spines and not hollow, 2 present as spines and hollow, 3 rodlet-like structures, not hollow |
|  | Pouch of gastric gizzard | 0 absent, 1 present |
|  | Oesophageal caecum | 0 absent, 1 present , 2 opening into gizzard |
|  | Caecum extending from stomach | 0 absent, 1 present without typhlosole, 2 present with typhlosole |
|  | Filter chamber | 0 absent, 1 present |
|  | Interior of stomach | 0 without cuticular lining, 1 with cuticular lining |
|  | Position of origin of intestine compared to opening of oesophagus into stomach | 0 intestine originated posterior from stomach (flow through system), 1 intestine opens next to oesophagus (U-shape) |
|  | Extent of intestinal typhlosole in intestine | 0 only in proximal part, 1 absent, 2 entire length of intestine |
|  | Hypo-/epiathroid circumoesophageal nerve ring | 0 epiathroid, 1 hypoathroid (pleural close to pedal ganglia), 2 all three ganglia are annexed |
|  | Visceral loop | 0 long, 1 short and annexed to the pedal and parapedal commissure |
|  | Nerve ring location | 0 prepharyngeal, 1 postpharyngeal |
|  | Distance between the cerebral ganglia (length of cerebral commissure) | 0 long, 1 short |
|  | Radular nerve | 0 separate, 1 fused |
|  | Gastrooesophageal ganglia | 0 absent, 1 present |
|  | Statocyst | 0 containing multiple otoconia, 1 containing one otolith |
|  | hermaphroditism | 0 absent, 1 present |
|  | Genital system | 0 monaulic, 1 androdiaulic, 2 triaulic  3 androdiaulic with completely separate genital systems, but ciliated interconnections between vas deferens and oviduct |
|  | External sperm groove | 0 present, 1 absent, 2 sunk into notum wall, 3 external and internal duct present, 4 external groove used for eggs transport |
|  | Gonad acini | 0 hermaphroditic acini, 1 separate male and female acini, 2 male and female acini completely separate |
|  | Copulatory organ | 0 non retractable, 1 retractable, 2 missing |
|  | Ejaculatory duct | 0 open groove along penis, 1 closed ejaculatory duct |
|  | Penial gland (prostatic gland annexed to penis) | 0 absent , 1 present |
|  | Position of penis or male opening | 0 far away from female genital opening, 1 near to female genital opening, 2 close to mouth |
|  | Allosperm vesicles (types) | 0 bursa copulatrix and receptaculum present, 1 only bursa present, 2 only receptaculum present, 3 allosperm bulb present (secretion and storage in one bulb), 4 novel bursa copulatrix, 5 bursa and receptaculum absent |
|  | Receptaculum seminis, location | 0 proximal, 1 distal, 2 joined with bursa |
|  | Bursa copuatrix, location | 0 distal, 1 proximal, N coded in previous character |
|  | Atrial gland | 0 absent, 1 present |
|  | Spermform in ampulla | 0 elongate, 1 round |
|  | Location of pericardial complex | 0 anterior or median, 1 posterior |
|  | Pulmonary vessels | 0 absent, 1 present |
|  | Connection between auricle and gill | 0 short, 1 long |
|  | Position of ventricle to atrium | 0 behind atrium, 1 left of atrium, 2 in front of atrium, 3 more to the right, 4 more to the left |
|  | Blood gland | 0 absent, 1 present |
|  | Location of blood gland | 0 adjacent to heart, 1 next or on top of nervous system |
|  | Renopericardial duct | 0 simple, 1 syrinx |
|  | Cnidosacs | 0 absent, 1 present |
|  | Prebranchial pocket (= prebranchial gland) | 0 absent, 1 present |
|  | Hypobranchial gland | 0 present, 1 absent |
|  | Purple gland (gland of Blochmann) | 0 absent, 1 present |
|  | Opaline gland (gland of Bohadsch) | 0 absent, 1 present |
|  | Pallial gland (*Runcina*) | 0 absent, 1 present |
|  | Marginal glands | 0 absent, 1 present |
|  | Gill glands | 0 absent, 1 present |
|  | Specialized vacuolated epithelium | 0 absent, 1 present |
|  | Diaphragma | 0 present, 1 absent |
|  | Spicules | 0 absent, 1 present |
|  | Duct between shell cavity and outside | 0 absent, 1 present |
|  | Hook sacs and buccal cones | 0 absent, 1 present |
|  | Mediodorsal bodies | 0 absent, 1 present |
|  | Rhinophoral nerve | 0 absent, 1 present |
|  | Nervus tentacularis | 0 present, 1 absent |
|  | Glandular stripe | 0 absent, 1 present |
